# Safety, tolerability, pharmacokinetics, and activity of the novel long-acting antimalarial DSM265: a two-part first-in-human phase 1a/1b randomised study

**DOI:** 10.1016/S1473-3099(17)30171-8

**Published:** 2017-06

**Authors:** James S McCarthy, Julie Lotharius, Thomas Rückle, Stephan Chalon, Margaret A Phillips, Suzanne Elliott, Silvana Sekuloski, Paul Griffin, Caroline L Ng, David A Fidock, Louise Marquart, Noelle S Williams, Nathalie Gobeau, Lidiya Bebrevska, Maria Rosario, Kennan Marsh, Jörg J Möhrle

**Affiliations:** a QIMR Berghofer Medical Research Institute, Brisbane, QLD, Australia; b Medicines for Malaria Venture, Geneva, Switzerland; c University of Texas Southwestern Medical Center, Dallas, TX, USA; d Q-Pharm Pty Ltd, Herston, QLD, Australia; e Columbia University, New York, NY, USA; f Takeda Development Center Americas, Inc, Cambridge, MA, USA; g AbbVie, North Chicago, IL, USA

## Abstract

**Background:**

DSM265 is a novel antimalarial that inhibits plasmodial dihydroorotate dehydrogenase, an enzyme essential for pyrimidine biosynthesis. We investigated the safety, tolerability, and pharmacokinetics of DSM265, and tested its antimalarial activity.

**Methods:**

Healthy participants aged 18–55 years were enrolled in a two-part study: part 1, a single ascending dose (25–1200 mg), double-blind, randomised, placebo-controlled study, and part 2, an open-label, randomised, active-comparator controlled study, in which participants were inoculated with *Plasmodium falciparum* induced blood-stage malaria (IBSM) and treated with DSM265 (150 mg) or mefloquine (10 mg/kg). Primary endpoints were DSM265 safety, tolerability, and pharmacokinetics. Randomisation lists were created using a validated, automated system. Both parts were registered with the Australian New Zealand Clinical Trials Registry, number ACTRN12613000522718 (part 1) and number ACTRN12613000527763 (part 2).

**Findings:**

In part 1, 73 participants were enrolled between April 12, 2013, and July 14, 2015 (DSM265, n=55; placebo, n=18). In part 2, nine participants were enrolled between Sept 30 and Nov 25, 2013 (150 mg DSM265, n=7; 10 mg/kg mefloquine, n=2). In part 1, 117 adverse events were reported; no drug-related serious or severe events were reported. The most common drug-related adverse event was headache. The mean DSM265 peak plasma concentration (C_max_) ranged between 1310 ng/mL and 34 800 ng/mL and was reached in a median time (t_max_) between 1·5 h and 4 h, with a mean elimination half-life between 86 h and 118 h. In part 2, the log_10_ parasite reduction ratio at 48 h in the DSM265 (150 mg) group was 1·55 (95% CI 1·42–1·67) and in the mefloquine (10 mg/kg) group was 2·34 (2·17–2·52), corresponding to a parasite clearance half-life of 9·4 h (8·7–10·2) and 6·2 h (5·7–6·7), respectively. The median minimum inhibitory concentration of DSM265 in blood was estimated as 1040 ng/mL (range 552–1500), resulting in a predicted single efficacious dose of 340 mg. Parasite clearance was significantly faster in participants who received mefloquine than in participants who received DSM265 (p<0·0001).

**Interpretation:**

The good safety profile, long elimination half-life, and antimalarial effect of DSM265 supports its development as a partner drug in a single-dose antimalarial combination treatment.

**Funding:**

Wellcome Trust, UK Department for International Development, Global Health Innovative Technology Fund, Bill & Melinda Gates Foundation.

## Introduction

Over the last 15 years the number of deaths from malaria has decreased by 63% globally as a result of better access to medicines and insecticide-treated bednets.^1^ This success has led to more ambitious goals, with a call from WHO to reduce morbidity and mortality by 90% over the next 15 years, moving towards eradication of malaria. The progress achieved towards the elimination of malaria is challenged by the development and spread of resistance. During the past decades, fixed-dose artemisinin combination therapies, recommended by WHO to be given for at least 3 days, have become the first-line treatment for uncomplicated malaria. Unfortunately, resistance against artemisinin and its partner drugs is developing in the Greater Mekong subregion of southeast Asia,^2^ with reports of multidrug-resistant strains.^3^, ^4^, ^5^, ^6^ New compounds are urgently required as potential new therapeutics.

The target product profile of an antimalarial drug is a combination therapy that would increase compliance, potentially allowing directly observed therapy, and prevent transmission.^7^ This profile would imply shortening the course of therapy from 3 days preferably to a single dose. Any new combination therapy should be composed of molecules with different modes of action, different resistance mechanisms, and complementary pharmacokinetics. In recent years, several new classes of antimalarials have entered clinical studies in malaria patients. These include the fast-acting agents artefenomel (OZ439),^8^ cipargamin (KAE609),^9^ and KAF156,^10^ whereas ferroquine remains the only long-acting novel antimalarial in clinical development.^11^, ^12^ Therefore, a need exists for additional long-acting molecules.

**Research in context**

**Evidence before this study**

Although the incidence of malaria has declined drastically in the last 15 years, WHO estimated that in 2015 there were more than 200 million cases worldwide, resulting in 429 000 deaths. The WHO Global Technical Strategy goal is to reduce malaria case incidence and mortality rates by 90% by 2030. To achieve this, new treatments for malaria will be needed. Additionally, the emergence of parasite resistance to current first-line antimalarials is threatening progress towards malaria elimination. To reduce the development of resistance, new treatments for malaria formulated as combination therapies are required. Such combinations should include components with different, and preferably novel, mechanisms of action. DSM265 is a new antimalarial candidate that was discovered through a target-based research strategy aimed at inhibiting *Plasmodium* dihydroorotate dehydrogenase (DHODH), an essential enzyme for pyrimidine biosynthesis.

We searched PubMed between Jan 1, 2013, and Dec 1, 2016, using the terms “dihydroorotate dehydrogenase plasmodium inhibitor” and “DSM265”, and the clinical trials registries ClinicalTrials.gov and the Australian New Zealand Clinical Trials Registry (ANZCTR). DSM265 is currently the first DHODH plasmodium inhibitor in clinical development for the treatment of malaria. Preclinical studies predicted a safety profile for DSM265 supporting human studies, as well as a long half-life in human beings. The in-vitro antiplasmodial activity of DSM265 was comparable with mefloquine and chloroquine. Recently, further clinical trials have been done to test the antimalarial activity of DSM265, including a study to assess its chemoprophylactic activity and a phase 2 study in clinical malaria.

**Added value of this study**

Integration of a malaria challenge cohort within a phase 1 human study allowed concurrent assessment of DSM265 safety, pharmacokinetics, and antimalarial activity. Our results show that DSM265 has a favourable safety profile in human beings, and a pharmacokinetic profile characterised by a long half-life. Plasma concentrations remained above the *P falciparum* minimum inhibitory concentration for more than 8 days, suggesting that DSM265 could potentially result in a single-dose cure when used with a partner drug. Additionally, pharmacodynamic analysis showed that DSM265 acts slowly against *P falciparum*. Therefore, DSM265 could be combined with a fast-acting drug to develop an antimalarial combination therapy.

**Implications of all the available evidence**

The design of this study allowed rapid determination of the key pharmacokinetic and pharmacodynamic parameters of DSM265, and estimated an efficacious dose for clinical malaria. This information, together with safety data, was crucial to enable the progression to a phase 2 study with malaria patients in Peru, accelerating the clinical development of DSM265. This phase 2 trial has already been completed and will be published shortly.

This study showed that DSM265 is a promising drug combination partner for single-dose treatment of acute uncomplicated malaria. A single-dose treatment would improve patient compliance compared with the current first line of treatment, which requires multiple daily dosing, and would therefore result in a better treatment outcome.

Dihydroorotate dehydrogenase (DHODH) is essential for pyrimidine biosynthesis in plasmodia because they lack pyrimidine salvage pathways and rely entirely on de-novo synthesis of pyrimidines. DSM265 was discovered through a target-based research strategy to develop an inhibitor of the plasmodial DHODH with high selectivity compared with the human orthologue.^13^ Preclinical studies predicted that DSM265 would have a safety profile to enable study in human beings, and that a single dose of DSM265 could potentially maintain plasma concentrations above the minimum inhibitory concentration (MIC) for over 8 days. In-vitro efficacy studies show that the DSM265 antiparasitic activity is achieved with a similar kill rate to atovaquone and pyrimethamine. In the *Plasmodium falciparum*-infected severe combined immunodeficient (SCID) mouse model, DSM265 had potent in-vivo antimalarial activity with 90% effective dose (ED_90_) of 3 mg/kg per day, comparing favourably with chloroquine (ED_90_=4·3 mg/kg) and mefloquine (ED_90_=7·7 mg/kg).^13^ A primary metabolite of DSM265, DSM450, also inhibits *P falciparum* DHODH, but shows much less antimalarial activity than DSM265.^13^

We undertook this first-in-human study to assess the safety, tolerability, and pharmacokinetic profile of DSM265 and its metabolite DSM450. A standard phase 1 safety and pharmacokinetic study protocol was integrated with an induced blood-stage malaria (IBSM) cohort, a controlled human malaria infection whereby healthy participants were inoculated with *P falciparum*-infected erythrocytes for preliminary assessment of antiparasitic activity.^14^ Using this approach, pharmacokinetic and pharmacodynamic parameters can be determined rapidly, helping both to assess whether the drug should be developed further, and to select doses for subsequent phase 2 efficacy studies in patients.

## Methods

### Study design and participants

This study consisted of two parts. Part 1 was a phase 1a, single ascending dose, double-blind, randomised, placebo-controlled study. Part 2 was a phase 1b, IBSM, open-label, randomised, active-comparator controlled study, in which participants were inoculated with blood-stage *P falciparum* and treated with a single dose of DSM265 or mefloquine. Preclinical efficacy studies done in the SCID mouse model estimated the plasma minimum parasiticidal concentration for DSM265 to be in the range of 1000–2000 ng/mL.^13^ Thus, when a single dose of DSM265 reached the targeted human exposure in part 1, and this dose was shown to be safe, part 2 was initiated. Both parts were done at Q-Pharm (Brisbane, QLD, Australia).

Healthy men and women (of non-childbearing potential) aged 18–55 years were eligible for the study. Participants were excluded if they had hyper-sensitivity to any study drug. In part 2, participants were not to live alone for the duration of the study, not to have had visited a malaria-endemic area for a period greater than 2 weeks in the last 12 months, and not to have received recent or current therapy with an antibiotic or drug with potential antimalarial activity. Full inclusion and exclusion criteria are listed in the appendix (pp 3–5). All participants gave written informed consent before being included in the study. The study was approved by the QIMR Berghofer Human Research Ethics Committee.

### Randomisation and masking

Randomisation lists for parts 1 and 2 were created using a validated, automated system. Participants in part 1 were randomly assigned to receive either a single dose of DSM265 or placebo. The DSM265 to placebo allocation ratio was: 2:1 in the 25 mg sentinel sub-cohort, 4:1 in the remainder of participants in this cohort, 8:2 in the 250 mg cohort (fasted–fed), and 6:2 in the other cohorts (75–150 mg and 400–1200 mg). Participants and investigators were masked to group allocation. Treatment identity was concealed by providing placebo and drug doses in identical packaging and appearance.

Part 2 took place in two subgroups, part 2a and 2b. Participants in part 2 were randomly assigned to receive either a single dose of DSM265 or treatment with mefloquine at a ratio 4:1 in part 2a and 3:1 in part 2b. Masking was not possible because of the different formats of the drugs given. However, the laboratory where parasite quantification assays by quantitative PCR (qPCR) were undertaken was masked to allocation.

### Procedures

In part 1, single ascending doses of DSM265 (25–1200 mg) or placebo were given to participants, randomised in eight dose cohorts. A starting dose of 25 mg was chosen in agreement with guidelines on dose selection for first-in-human studies based on preclinical safety data,^15^, ^16^ which set a safety cap for exposure at 2985 μg·h/mL. In this first 25 mg cohort, a sentinel sub-cohort (n=3) was dosed on day 1. Safety and pharmacokinetic data were reviewed in a blinded fashion before dosing the rest of the cohort, and before dose escalation in the following cohorts. In the following cohorts, all participants were dosed on day 1. In the 250 mg dose cohort, participants were dosed in a fasted state and returned 21 days after the first DSM265 dose to receive treatment after consumption of a US Food and Drug Administration standard high-fat breakfast.^17^ Participants in all other cohorts received DSM265 after a fasting period of at least 8 h. Participants were followed up for 21 days after DSM265 dosing, except for participants in the 1200 mg cohort, who were followed up for 35 days due to the long elimination half-life of the metabolite DSM450.

DSM265 (WuXi AppTec Co, Ltd, Shanghai, China) was supplied as a 25% (250 mg/g) spray-dried dispersion of the active pharmaceutical ingredient in hydroxypropylmethylcellulose acetate succinate (HPMCAS-MF) as powder in a bottle (Bend Research Inc, Bend, OR, USA). The powder was suspended in vehicle (0·1% methocel A4M, 0·1% polysorbate 80, 0·005% simethicone, 0·05% ethyl vanillin, and 0·5% sucralose; 240 mL for 25–400 mg doses, 340 mL for 600–800 mg doses, and 400 mL for the 1200 mg dose), and given as an oral suspension on site. Plasma concentrations during the absorption phase of DSM265 in the 400 mg cohort showed lower exposure than expected due to incomplete dispersion of the drug. Thus, the preparation instructions were modified to ensure adequate product dispersion, and the 400 mg cohort was repeated, with subsequent dose preparation using the amended instructions. The placebo (Shin-Etsu Chemical Company, Ltd, Tokyo, Japan) was supplied as HPMCAS-MF as powder in a bottle, and suspended according to the same protocol.

In part 2, an IBSM cohort was inoculated as described previously.^14^ Briefly, participants were inoculated intravenously on day 0 with *P falciparum*-infected human erythrocytes (about 1800 viable parasites). Parasite growth was monitored by qPCR targeting 18S DNA.^18^ The threshold for treatment was when all participants reached parasitaemia levels of at least 800 parasites per mL, or earlier if any participant reached at least 2000 parasites per mL or clinical evidence of malaria occurred. Fasted participants received either DSM265 (150 mg) or mefloquine (10 mg/kg; Lariam; Roche Products Pty Ltd, Dee Why, NSW, Australia). Participants in the DSM265 group were to receive curative treatment with artemether-lumefantrine (Riamet; Novartis Pharmaceuticals Pty Ltd, Macquarie Park, NSW, Australia) 21 days after DSM265 treatment (day 29), or earlier if recrudescence occurred. If participants remained gametocytaemic at the end of the study, they were treated with 45 mg primaquine (Primacin; BNM Group, Sydney, NSW, Australia). Gametocytaemia was monitored using quantitative reverse transcriptase PCR (qRT-PCR) targeting *pfs25* mRNA,^19^ a transcript expressed in mature female gametocytes.^20^

Safety assessment was done at screening and at protocol-specified times (appendix pp 6–10). Safety parameters included adverse events by spontaneous reporting and non-directive questioning of the participant at each visit, physical examination, vital signs, clinical laboratory evaluation, electrocardiogram (triplicate ECGs), and cardiac telemetry up to 24 h post-dose.

Blood samples to determine concentrations of DSM265 and its major metabolite DSM450 were taken before DSM265 dosing and at 0·5, 1, 2, 4, 6, 8, 12, 24, 48, 96, 144, 216, 312, and 480 h post-dosing. To adequately capture exposure in the 1200 mg cohort, two extra pharmacokinetic timepoints were included in this cohort at 648 h and 816 h post-dosing. The pharmacokinetic profile of DSM450 was determined from the 150 mg cohort onward. Blood and plasma samples were analysed by liquid chromatography-tandem mass spectrometry (HPLC-MS/MS; appendix pp 11–12).

Parasitaemia was measured each morning from day 4 until qPCR results became positive, and thereafter at 12 h intervals until treatment; before DSM265 dosing, and at 4, 8, 12, 16, 20, 24, 30, 36, 48, 60, 72, 84, 96, and 108 h post-dosing; subsequent measurements were about three times per week until two consecutive negative results were observed. Gametocytaemia was measured on days 12–32, and at the end of study.

To study the potential of DSM265 to inhibit the activity of human DHODH, concentrations of biochemical products of DHODH activity (uridine and uridine nucleotides) were measured by HPLC-MS/MS (appendix p 13) as exploratory biomarkers in blood of participants in the 800 mg (0, 4, 6, 12, 24, and 96 h post-treatment) and 1200 mg (−0·5, −0·25, 0, 4, 8, 12, 24, and 96 h post-treatment) cohorts.

The induction of *P falciparum dhodh* (*pfdhodh*) genetic modifications that could confer DSM265 drug resistance was investigated in any recrudescent parasite population. Blood was taken from participants in part 2 at the time of recrudescence. Parasite DNA was extracted from blood samples, the *pfdhodh* genes were amplified by PCR, and their DNA sequence determined (appendix p 14).

### Outcomes

The primary endpoints were safety and the pharmacokinetic profile of single ascending doses of DSM265, as well as establishing the maximum tolerated dose (MTD) in healthy participants. The pharmacokinetic parameters determined were maximum blood or plasma concentration (C_max_), timepoint when C_max_ was reached (t_max_), maximum blood or plasma concentration 7 days after drug administration (C_168h_), area under the concentration–time curve from 0 h extrapolated to infinity (AUC_0–∞_), and elimination half-life (t_1/2_).

Secondary endpoints were the logarithm of the parasite reduction ratio per 48 h (log_10_PRR_48_) and parasite clearance half-life, assessment of the effect of food on DSM265 pharmacokinetics and tolerability, and characterisation of the pharmacokinetics of DSM450.

### Statistical analysis

To test the proportionality between DSM265 concentrations in blood and plasma in part 1, a power model was fitted to the data in the log-transformed form, ln(y)=ln(a) + b × ln(x), where ln(a) is the intercept and b is the slope. To estimate the proportionality coefficient, a proportional model was fitted to the data in the log-transform form, ln(a)=ln(y/x), where a is the proportionality coefficient. Phoenix WinNonlin version 6.3 was used for both models. Pharmacokinetic parameters were determined by non-compartmental analysis using Phoenix WinNonlin version 6.3 in part 1 and R version 3.1.1 in part 2. In part 1, population pharmacokinetic analysis was done using the NONMEM software and the non-linear mixed-effects modelling approach (appendix p 15); R version 3.0.2 was used for pharmacokinetic data exploration.

In part 2, log_10_PRR_48_ and parasite clearance half-life were estimated using the slope of the optimal fit for the log-linear relationship of the parasitaemia decay.^21^ Individual log_10_PRR_48_ and corresponding 95% CI were calculated using the slope and corresponding SE of the optimal regression model. The log_10_PRR_48_ and parasite clearance half-life for a dose cohort were derived using the weighted mean of the optimal slope for participants with an adequate model fit (p<0·001). An omnibus test was used to investigate differences in log_10_PRR_48_ between groups. R version 3.0.2 was used for analysis of parasite clearance. The timepoint at which the parasitaemia nadir occurred was estimated from individual log-linear parasite clearance curves. The DSM265 concentration at nadir was assigned as the MIC. Single-dose pharmacokinetic profiles of DSM265 were simulated by the population pharmacokinetic model developed with part 1 pharmacokinetic data to identify a single curative dose, defined as a dose that would maintain DSM265 concentration above the maximum estimated MIC for a minimum of 7 days.

In part 1, the difference in concentrations of uridine and uridine nucleotides from timepoint 0 h to 96 h between treatment groups was analysed by a two-sample t test. Differences in changes over time between treatment groups were determined by a linear mixed model with main effects and an interaction for time and treatment group. Analyses were stratified by DSM265 cohorts 800 mg and 1200 mg. All analyses were done in Stata version 13 (5% significance level).

Previous IBSM studies have determined, based on two-sided *t* tests at the 5% significance level, that a cohort size of eight would identify a difference of 25% in the parasite clearance rate with 80% power. Mefloquine control participants were not included in sample size calculations because their purpose was to observe parasite growth and clearance curves in response to a drug with known activity.

The studies were registered with the Australian New Zealand Clinical Trials Registry as number ACTRN12613000522718 (part 1) and number ACTRN12613000527763 (part 2).

### Role of the funding source

The funders of this study had no role in study design, data collection, analysis, and interpretation or reporting. The corresponding authors had access to all data in the study and had final responsibility for the decision to submit for publication.

## Results

**Figure 1:**
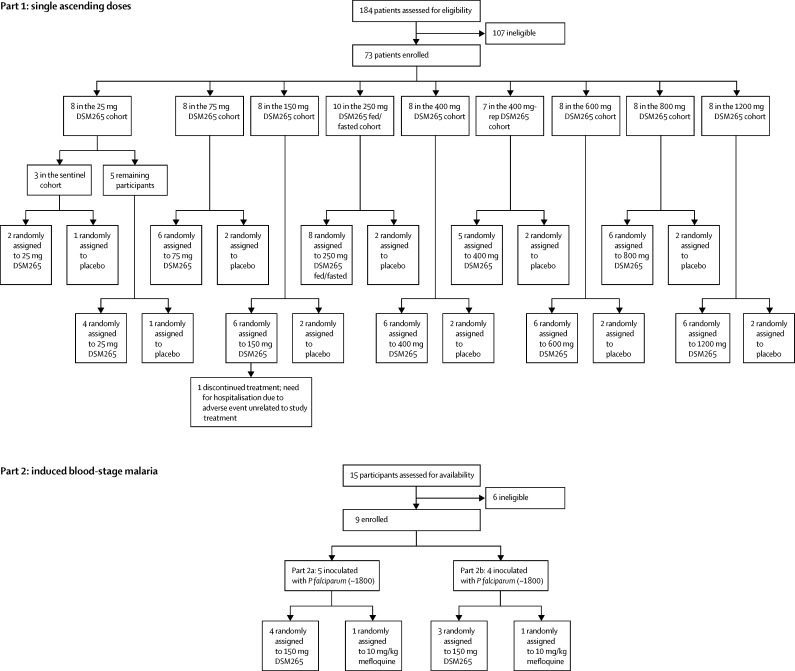
Trial profile In part 1, single ascending doses of DSM265 (25–1200 mg) were tested in eight cohorts in fasted conditions. Participants in the 250 mg cohort were to return at least 21 days after the first DSM265 dose to receive DSM265 in fed conditions. The 400 mg dose cohort was repeated (400 mg-rep) due to a biopharmaceutical issue in the preparation of the study medication. Part 2 (induced blood-stage malaria) started after documentation of safety and pharmacokinetics data of the 150 mg dose in part 1.

In part 1, 73 participants were enrolled and randomly assigned in eight cohorts to receive either a single dose of DSM265 (25–1200 mg, n=55) or placebo (n=18; figure 1). Part 1 took place between April 12, 2013, and July 14, 2015. In part 2, nine participants were enrolled, inoculated with *P falciparum*, and treated on day 8 with either DSM265 or mefloquine (figure 1). Part 2 was done in two groups between Sept 30 and Nov 25, 2013 (part 2a) and between Oct 15 and Dec 10, 2013 (part 2b). One participant in part 1 (150 mg cohort) withdrew from the study on day 17 because of a serious adverse event unrelated to study treatments. The remaining participants completed the study. All participants were included in the outcome analysis. Baseline characteristics of participants are presented in table 1.

**Table 1:** Baseline characteristics

		**Part 1: single ascending doses**			**Part 2: IBSM**		
		DSM265 (n=55)	Placebo (n=18)	Total (n=73)	DSM265 (n=7)	Mefloquine (n=2)	Total (n=9)
Age (years)		26·1 (6·8)	27·9 (8·9)	26·6 (7·3)	24·1 (2·0)	27·0 (5·7)	24·8 (2·9)
Race							
	White	47 (85%)	15 (83%)	62 (83%)	7 (100%)	1 (50%)	8 (89%)
	Asian	6 (11%)	1 (6%)	7 (10%)	0	1 (50%)	1 (11%)
	Black African	2 (4%)	0	2 (3%)	0	0	0
	Pacific Islander	0	2 (11%)	2 (3%)	0	0	0
Height (cm)		177·9 (6·4)	179·6 (6·1)	178·3 (6·4)	175·0 (8·3)	181·0 (7·1)	176·3 (8·1)
Bodyweight (kg)		78·7 (11·1)	79·1 (11·2)	78·8 (11·0)	73·5 (10·8)	77·3 (8·3)	74·4 (10·0)
Body-mass index (kg/m^2^)		24·8 (3·0)	24·5 (3·0)	24·8 (3·0)	23·9 (2·4)	23·6 (0·6)	23·8 (2·1)

Data are n (%) or mean (SD). All participants were male. IBSM=induced blood-stage malaria.

DSM265 had a good safety and tolerability profile at all the doses tested, thus a formal MTD could not be determined. In part 1, 117 adverse events were reported, most of them mild in severity (grade 1; table 2, appendix pp 16–20). Two serious adverse events were reported in two participants; both were deemed not related to study treatments: one, an accident causing multiple injuries in the 150 mg cohort; the other a case of Bell's palsy, in the 75 mg cohort, which was deemed not to be drug-related after consultation with a neurologist. The incidence of participants reporting total adverse events and drug-related adverse events was higher in the DSM265 than in the placebo group, but did not increase with ascending doses (table 2). The most common drug-related adverse event reported was headache, which had a higher incidence in participants in the DSM265 than in the placebo group (seven [13%] of 55 *vs* one [6%] of 18; relative risk 2·3 [95% CI 0·42–14·0], p=0·67). Other drug-related adverse events included dry mouth, malaise, nausea, vomiting, decreased appetite, thrombocytopenia, and increased reticulocyte counts (appendix p 21). Each of these adverse events was reported only once during the study.

**Table 2:** Adverse events after administration of single ascending doses of DSM265 by dose cohort (n=73)

	**DSM265 dose**									**Total DSM265 (n=55)**	**Placebo (n=18)**	**Total (n=73)**
	25 mg (n=6)	75 mg (n=6)	150 mg (n=6)	250 mg (fasted; n=8)	250 mg (fed; n=8)	400 mg (n=11)	600 mg (n=6)	800 mg (n=6)	1200 mg (n=6)			
**Number of participants with adverse events**												
Participants with adverse events	6 (100%)	3 (50%)	5 (83%)	8 (100%)	7 (88%)	5 (45%)	3 (50%)	3 (50%)	5 (83%)	38 (69%)	7 (39%)	45 (62%)
Participants with study drug related adverse events	0	1 (17%)	1 (17%)	3 (38%)	1 (13%)	2 (18%)	1 (17%)	0	3 (50%)	11 (20%)	1 (6%)	12 (16%)
Participants with grade 2–4 adverse events	3 (50%)	1 (17%)	1 (17%)	2 (25%)	1 (13%)	2 (18%)	2 (33%)	0	4 (67%)	15 (27%)	1 (6%)	16 (22%)
Participants with study drug related grade 2–4 adverse events	0	1 (17%)	0	0	0	2 (18%)	1 (17%)	0	1 (17%)	5 (9%)	0	5 (7%)
Participants with serious adverse events	0	1 (17%)	1 (17%)	0	0	0	0	0	0	2 (4%)	0	2 (3%)
**Number of events**												
Adverse events	12	12	9	14	15	15	6	5	14	102	15	117
Study drug related adverse events	0	1	1	3	1	4	1	0	4	15	1	16
Grade 2–4 adverse events	4	9	1	2	1	3	2	0	7	29	1	30
Study drug related grade 2–4 adverse events	0	1	0	0	0	3	1	0	2	7	0	7
Serious adverse events	0	1	1	0	0	0	0	0	0	2	0	2

The Common Terminology Criteria for Adverse Events (CTCAE 4.03) was used to grade adverse events (grade 1-5). The 400 mg dose combines participants from the two cohorts treated with this dose (n=6 and n=5 for the first and second cohort, respectively). No serious adverse event was deemed related to the study drug.

In part 1, two cases of thrombocytopenia were reported after administration of DSM265 (400 mg and 1200 mg). The lowest platelet count (78 × 10^9^ per L; normal range 150–400 × 10^9^ per L) was detected on day 5 in one participant in the 1200 mg cohort, and was suspected to be drug-related. This participant presented a non-clinically significant decrease in haemoglobin (from 137 g/L at baseline to 122 g/L 10 days after DSM265 dosing), and a clinically significant elevated reticulocyte count on days 10 and 14 (135 × 10^9^ per L and 174 × 10^9^ per L, respectively; normal range 10–100 × 10^9^ per L); the elevated reticulocyte was suspected to be drug-related. This participant was diagnosed with a haemoglobinopathy (sickle cell trait), unknown to the investigators at study entry. Review of individual reticulocyte count and haptoglobin concentrations of participants across all dose cohorts did not reveal any other clinically significant changes.

Two cases of isolated and asymptomatic aminotransferase increase were reported in part 1, both not considered as drug-related. One participant (400 mg) showed an increase in alanine aminotransferase (116 U/L, normal range 5–40 U/L), and another participant (250 mg, fed conditions) had an increase in aspartate aminotransferase (115 U/L, normal range 10–40 U/L). No cardiac arrhythmias or increase in QTc were observed up to the highest DSM265 dose tested.

In part 2, eight of the nine participants reported at least one adverse event. Of the 30 adverse events reported, 18 were deemed as probably related to malaria (appendix p 22). There were no adverse events related to DSM265 or any other study treatments, including mefloquine. Most of the adverse events were mild in severity (28 [93%] of 30). The most common adverse events deemed related to malaria were headache (n=8 participants) and fever (n=5 participants). No clinically significant changes in laboratory parameters (including white-blood-cell count, platelets, and liver function tests), ECGs, or cardiac telemetry were reported in the IBSM cohort.

**Figure 2:**
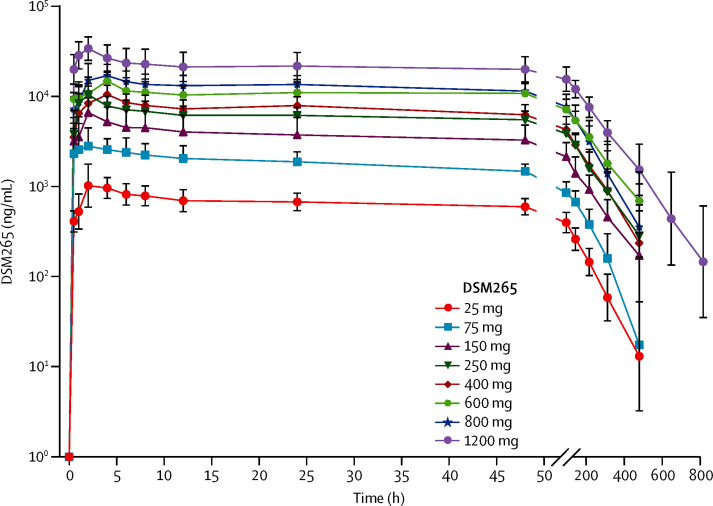
DSM265 plasma concentration versus time profiles under fasted conditions Geometric mean plasma concentrations of participants in different dose cohorts following administration of single doses of DSM265. Time 0 h corresponds to time of drug administration. For the 400 mg cohort, only data for the repeated cohort are represented. For all cohorts, n=6, except for the 250 mg dose cohort (n=8) and the 400 mg dose cohort (n=5). Error bars represent SDs.

The concentration of DSM265 measured in blood and plasma showed a proportional relationship; DSM265 concentrations in plasma were almost double that in blood (table 3, appendix p 23). This proportionality was confirmed by the estimated slope in the power model (b=0·98, 95% CI 0·98–0·99), and an estimated mean proportionality coefficient of 0·52 (0·51–0·53). Plasma pharmacokinetic analysis of DSM265 single doses tested in fasted conditions showed that the mean C_max_ increased with dose (range 1310–34 800 ng/mL) in a less than dose-proportional manner (figure 2, table 3). Similar inter-participant variability was observed in C_max_ across dose cohorts (coefficient of variation [CV%] range 22–40%). C_max_ was reached in a median range between 1·5 h and 4 h (t_max_). C_168h_ increased in an approximately dose-proportional manner (range 217–10 200 ng/mL). Likewise, plasma AUC_0–∞_ increased with dose (range 107 000–4 720 000 ng·h/mL) in an approximately dose-proportional manner between 25 mg and 250 mg, and then between 250 mg and 1200 mg, except the 800 mg dose, where sub-proportionality was observed. Inter-participant variability in AUC_0–∞_ was similar across dose cohorts (CV% range 18–39%). DSM265 showed a long elimination half-life, ranging from 86 h to 118 h across the doses tested. Assessment of the effect of food in the pharmacokinetic profile of DSM265 (250 mg) indicated that mean C_max_ decreased with food intake and t_max_ increased (appendix pp 24–25), whereas C_168h_ and AUC_0–∞_ were not affected. Overall, the effect of food on the pharmacokinetic profile of this DSM265 formulation was not considered clinically relevant at the dose tested. The C_max_ of DSM450 was reached later than for DSM265 (appendix p 26). The ratio of exposure of DSM450 to DSM265 ranged between 19% and 27% across cohorts, which is within the range observed in preclinical studies.

**Table 3:** Plasma pharmacokinetic variables of DSM265 according to dose cohort

	**C_max_ (ng/mL)**	**t_max_ (h)**	**C_168h_ (ng/mL)**	**AUC_480_ (h·ng/mL)**	**AUC_0–∞_ (h·ng/mL)**	**t_1/2_ (h)**
25 mg (n=6)	1310 (32)	2 (1–4)	217 (30)	104 000 (22)	107 000 (22)	88 (25)
75 mg (n=6)	3850 (40)	1·5 (0·5–4)	536 (35)	277 000 (21)	284 000 (22)	86 (28)
150 mg (n=6)	6630 (39)	2 (2–4)	1230 (41)	598 000 (39)	624 000 (39)	103 (19)
250 mg (n=8)	11 900 (28)	2 (1–2)	2380 (29)	1 070 000 (24)	1 130 000 (25)	104 (27)
400 mg (n=5)	11 500 (28)	4 (2–4)	2500 (36)	1 160 000 (32)	1 210 000 (36)	96 (28)
600 mg (n=6)	15 500 (22)	4 (2–4)	4620 (27)	2 010 000 (25)	2 140 000 (26)	114 (14)
800 mg (n=6)	19 100 (25)	2 (2–4)	4460 (46)	2 110 000 (33)	2 220 000 (36)	93 (46)
1200 mg (n=6)	34 800 (28)	2 (2–4)	10 200 (21)	4 310 000 (20)	4 720 000 (18)	118 (45)

Data are geometric means (coefficient of variation) except median (range) for t_max_. For the 400 mg dose cohort, only results of the repeated dose cohort are presented. C_max_=peak plasma concentration. t_max_=timepoint at which C_max_ is reached. C_168h_=DSM265 concentration 168 h post-dose. AUC_480_=area under the concentration–time curve from 0 h to 480 h post-dose. AUC_0–∞_=area under the concentration–time curve from 0 h to infinity. t_1/2_=estimated elimination phase half-life.

Population plasma pharmacokinetic analysis was done in participants from cohorts 25 mg to 800 mg (fasted conditions, n=44). The pharmacokinetic profile was best described by a two-compartmental model with zero-order absorption, dose-dependent duration of absorption and bioavailability, simple exponential independent random effect specification, and proportional residual variability (appendix pp 28, 29).

The DSM265 dose selected for the IBSM cohort in part 2 was 150 mg. The mean plasma C_168h_ for this dose in part 1 was 1230 ng/mL, which was at the lower end of the predicted efficacious concentration range determined in preclinical studies in plasma (1000–2000 ng/mL). Pharmacokinetic analysis of DSM265 from the seven participants in the IBSM cohort indicated that low blood-stage parasitaemia levels had no effect on the pharmacokinetic parameters (appendix p 27).

**Figure 3:**
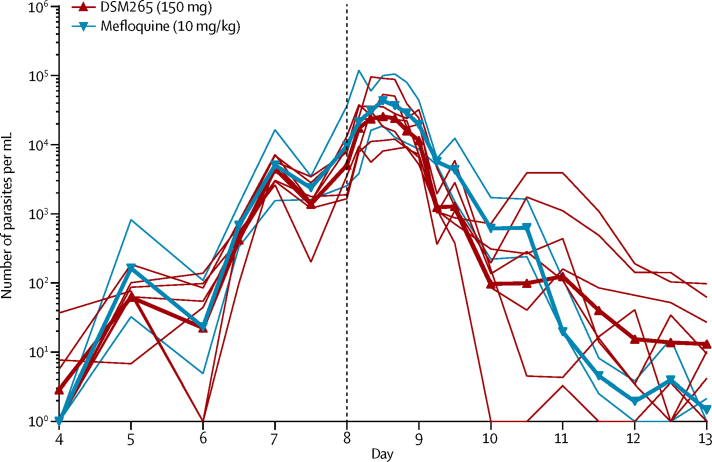
Parasite clearance profiles Participants were inoculated on day 0 and treated either with DSM265 (150 mg, red lines) or mefloquine (10 mg/kg, blue lines) on day 8 (vertical dashed line). Thin lines show individual curves and thick lines represent the mean.

Parasitaemia levels in participants in part 2 who received DSM265 showed an initial lag after treatment administration, which was followed by a first-order exponential decrease in parasitaemia (figure 3, appendix p 30). Median parasitaemia before treatment was 5773 parasites per mL (range 1676–36 633); recrudescence occurred in all seven participants between 10 and 22 days after DSM265 administration, when they were treated with artemether-lumefantrine. Parasitaemia levels in the two participants who received mefloquine decreased below the limit of detection of the qPCR assay (64 parasites per mL) 3 days after treatment without subsequent recrudescence. The presence of *pfs25* transcripts, which indicate gametocytaemia, were first detected 5 days after administration of DSM265 (day 13; appendix p 31). Three participants in the DSM265 treatment group received primaquine 37–39 days after inoculation.

Pharmacodynamic analysis of data from participants in part 2 dosed with DSM265 showed that the parasitaemia nadir was reached at a median time of 120 h (range 60–216), which corresponded to a median blood MIC of 1040 ng/mL (range 552–1500). Simulations in the population pharmacokinetic model developed with part 1 data predicted that a dose of 340 mg would maintain DSM265 blood concentrations above 1500 ng/mL, the maximum estimated MIC, for 7 days.

The dose-specific log_10_PRR_48_ estimated in participants with significant regression models of the log-linear relationship of the parasite decay in the DSM265 (150 mg, n=6) and mefloquine (10 mg/kg, n=2) groups were 1·55 (95% CI 1·42–1·67) and 2·34 (2·17–2·52), respectively, corresponding to a parasite clearance half-life of 9·4 h (8·7–10·2) and 6·2 h (5·7–6·7), respectively. Parasite clearance was significantly faster in participants who received mefloquine than in participants who received DSM265 (p<0·0001). Parasite clearance for participants in part 2a was significantly lower than in part 2b (p<0·0001; appendix pp 32–33).

DSM265 given at either 800 mg or 1200 mg did not significantly alter the concentrations of uridine or uridine nucleotides, either when the concentrations at 0 h were compared with those at 96 h after treatment, or when longitudinal changes over the first 96 h after treatment were compared with the placebo group (appendix pp 34–37).

DNA sequence analysis of the *pfdhodh* gene from parasite DNA isolated at the time of recrudescence (days 12–18) in five participants who received 150 mg DSM265 did not show mutations compared with the canonical 3D7A gene sequence.

## Discussion

In this study, we integrated for the first time the assessment of antimalarial activity of a drug within a first-in-human study, thereby accelerating the development of DSM265, the first plasmodium-selective DHODH inhibitor tested in human beings. The results show that DSM265 has a good safety profile and a long half-life, with a plasma concentration that remained above the MIC for more than 8 days. This prolonged effect against blood-stage *P falciparum* is crucial to the pharmacodynamics of DSM265, supporting its further clinical development as a component of a novel combination antimalarial therapy.

Although DSM265 showed a good safety profile, the drug-related thrombocytopenia and elevated reticulocytes reported in a participant diagnosed with sickle-cell trait will require further attention in patients with clinical malaria.

The elimination half-life of DSM265 was the longest of any of the new-generation antimalarials; however, it is still shorter than 4-aminoquinolines or aminoalcohols that have been tested in the IBSM model, such as mefloquine (10 mg/kg)^22^ or ferroquine (800 mg).^12^ Importantly, the pharmacokinetic parameters of DSM265 were not altered by food or low levels of blood-stage malaria infection. A mild decrease in C_max_ was observed when DSM265 was given with high-fat food but this effect was not clinically significant.

The activity of DSM265 against *P falciparum* was characterised by an initial lag phase, accompanied by an increase in parasitaemia immediately post-treatment. This increase is probably due to a combination effect of the drug's modest speed of action and the lifecycle stage at which the drug exposure occurred.^23^ When DSM265 was given (day 8), parasites were mainly in the mature stage and about to undergo schizogony. Thus, an increase in parasitaemia would happen in the following 12 h irrespective of treatment administration. Overall, the activity of DSM265 (150 mg) against blood-stage *P falciparum* was slower (log_10_PRR_48_ 1·55 [95% CI 1·42–1·67]) than other long-acting drugs tested in the IBSM model, such as mefloquine (10 mg/kg, log_10_PRR_48_ 2·20 [2·11–2·28])^22^ and ferroquine (800 mg, log_10_PRR_48_ 2·21 [2·15–2·27]).^12^ There was a statistically significant difference between the log_10_PRR_48_ of the two groups in part 2 (part 2a and 2b), which was not clinically significant, since both values were in the range characteristic of slow-acting drugs. The small size of the groups (n=3) might have contributed to the log_10_PRR_48_ variability between them. The log_10_PRR_48_ reported for mefloquine in this study is in accordance with previous reports, which validates the reproducibility of the IBSM model.^22^

The dose selected for the IBSM cohort resulted, after initial parasite clearance, in recrudescence in all participants, which allowed calculation of the MIC for DSM265. The design used in this study enabled both identification of the MIC and the prediction of the efficacious dose within 6 months from initiation of clinical development. Applying a conventional development process of separate phase 1 in healthy participants followed by phase 2a proof-of-concept studies in patients would take over 2 years. In the next step of DSM265 clinical development, it would be important to evaluate its efficacy in clinical malaria to confirm the pharmacokinetic and pharmacodynamic parameters. Data available from previous IBSM studies indicate that the pharmacodynamic parameters estimated in this study will correspond to those obtained in malaria patients.^22^ Furthermore, DSM265 could be combined with a fast-acting antimalarial drug in development to potentially find a single encounter cure.

Combining DSM265 with a partner drug would be beneficial not only from a pharmacodynamic point of view, but also to reduce the probability of developing drug resistance.^24^, ^25^ In preclinical studies drug resistance to DSM265 resulted either from point mutation or through *pfdhodh* gene amplification.^13^ However, no point mutations in the *pfdhodh* gene were identified in the IBSM cohort. *pfdhodh* gene amplification events could not be studied due to low parasitaemia. Antimalarial resistance to other antimetabolites, such as sulfadoxine–pyrimethamine and atovaquone, can be more readily induced in vitro and in vivo than for drugs with other mechanisms of action.^26^, ^27^ Monitoring for the emergence of drug resistance parasites will be required in future studies.

Generalisability of the study findings is limited by the recruitment of only men due to the contraceptive requirements of the study impeding recruitment of women. Evaluation in a wider population will be needed in further studies. Another limitation of this study was that the preparation of the DSM265 formulation was cumbersome, and drug reformulation will be required in future studies.

In conclusion, DSM265 is a novel antimalarial with a good safety profile. Its long half-life suggests that it is a promising drug combination partner for single-dose treatment of acute uncomplicated malaria. Treatment with single-dose DSM265 could improve patient compliance and thus treatment outcome compared with current treatment options, which require multiple daily dosing.
