# Supplementary material for: Safety, tolerability, pharmacokinetics, and activity of the novel long-acting antimalarial DSM265: a two-part first-in-human phase 1a/1b randomised study
Source: Lancet Infect Dis. 2017 Jun;17(6):626–35. doi: 10.1016/S1473-3099(17)30171-8 (PMC5446412; doi:10.1016/S1473-3099(17)30171-8)
Supplement: Supplementary appendix [file mmc1.pdf]

THE LANCET
Infectious Diseases

Supplementary appendix

This appendix formed part of the original submission and has been peer reviewed. We post it as supplied by the authors.

Supplement to: McCarthy JS, Lotharius J, Rückle T, et al. Safety, tolerability, pharmacokinetics, and activity of the novel long-acting antimalarial DSM265: a two-part first-in-human phase 1a/1b randomised study. *Lancet Infect Dis* 2017; published online March 28. http://dx.doi.org/10.1016/S1473-3099(17)30171-8.

Supplementary webappendix

Table of contents

Methods

Inclusion and exclusion criteria

Schedule of events

Table S1. Schedule of events part 1: single ascending dose

Table S2. Schedule of events part 2: IBSM

Determination of DSM265 levels in plasma and blood

Determination of DSM450 levels in plasma

Determination of uridine and uridine nucleotides

Assessment of drug resistance

Table S3. *Pfdhodh* amplification and **s**equencing primers

Population pharmacokinetic model

Results

Display of adverse events

Table S4. Summary of adverse events following administration of single-doses of DSM265 by system organ class, MedDRA preferred term and dose cohort.

Table S5. Summary of adverse events related to DSM265 treatment by dose cohort

Table S6. Summary of adverse events in the IBSM cohort

Table S7. Probably related adverse events in the IBSM cohort

Pharmacokinetics analysis

Table S8. Blood pharmacokinetic variables of DSM265 by dose cohort (fasted conditions)

Figure S1. Mean plasma concentration following administration of DSM265 (250 mg) under fasted and fed conditions

Table S9. Plasma pharmacokinetic variables of DSM265 dose cohort 250 mg in fasted and fed conditions

Table S10. Plasma pharmacokinetic variables of DSM450 by dose cohort

Table S11. Blood pharmacokinetic variables of DSM265 (150 mg) in part 1 (single ascending dose) and part 2 (IBSM)

Table S12. Population pharmacokinetic parameters of DMS265 in plasma

Figure S2. Population pharmacokinetic analysis visual predictive check by DMS265 dose cohort

Parasitaemia and gametocytaemia levels in the IBSM cohort

Figure S3. Parasite clearance profiles

Figure S4. Individual parasitaemia and gametocytaemia levels in the IBSM cohort following administration of DSM265 (150 mg)

Parasite reduction ratio in the IBSM cohort

Table S13. Log10PRR48 and parasite clearance half-lives

Table S14. Participant-specific $log_{10}PRR_{48}$ part 2a and 2b

Human DHODH activity

Figure S5. Uridine and uridine nucleotide levels after treatment with DSM265 (800 mg)

Figure S6. Uridine and uridine nucleotide levels after treatment with DSM265 (1200 mg)

Table S15. Comparison of changes in uridine and uridine nucleotides levels from 0 to 96 h between DSM265 (800 mg) and placebo groups

Table S16. Comparison of changes in uridine and uridine nucleotides levels from 0 to 96 h between DSM265 (1200 mg) and placebo groups

Table S17. Comparison of longitudinal changes in uridine and uridine nucleotides levels over time between DSM265 (800 mg) and placebo groups

Table S18. Comparison of longitudinal changes in uridine and uridine nucleotides levels over time between DSM265 (1200 mg) and placebo groups

References

Inclusion and exclusion criteria

Inclusion criteria

Participants eligible for inclusion in the study had to fulfil **all** of the following criteria:

1. Written informed consent had to be obtained before any assessment was performed.

2. Healthy male and female (of non-childbearing potential) participants 18–55 years of age in part 1, and 18–50 years of age in part 2, in good health as determined by past medical history, physical examination, vital signs, electrocardiogram (ECG), and laboratory tests at screening.

- Women were considered post-menopausal and not of child bearing potential if they had at least 12 months of natural (spontaneous) amenorrhea with an appropriate clinical profile (e.g., age appropriate, history of vasomotor symptoms) or 6 months of spontaneous amenorrhea with serum FSH levels at the local laboratory levels for post-menopause; or had surgical bilateral oophorectomy or bilateral salpingectomy (with or without hysterectomy) at least 6 months before the study. Women on oral contraceptives were to be excluded from the study, and also women who had a tubal ligation.

3. At screening and baseline, vital signs (systolic and diastolic blood pressure and pulse rate) were to be assessed in the sitting position after the participant had rested for at least 3 min and again when required after 3 min in the standing position. Investigators could be guided by the following ranges:

- oral body temperature, 35·0–37·5°C
- systolic blood pressure, 90–140 mm Hg
- diastolic blood pressure, 50–90 mm Hg
- pulse rate, 40–90 bpm.

4. When blood pressure and pulse were taken after at least 3 min standing, there should be no more than a 20 mm Hg drop in systolic or 10 mm Hg drop in diastolic blood pressure and increase in heart rate (>20 bpm) compared to the sitting results. Higher increase is associated with clinical manifestation of postural hypotension. Any participant exhibiting clinical manifestations of postural hypotension was to be excluded.

5. Had to have haematology, clinical chemistry and urinalysis results at screening that were within the reference range or, if outside the range, not clinically significant as judged by the investigator and confirmed and agreed by the medical monitor; aspartate aminotransferase (AST), alanine aminotransferase (ALT), and bilirubin must have been within the reference range at screening.

6. Participants had to:

- weigh at least 50 kg to participate in the study, and had to have a body mass index (BMI) within the range of 18–32 kg/m^2
- be able to communicate well with the investigator, to understand and comply with the requirements of the study.

7. Had good peripheral venous access.

Exclusion criteria

Participants who fulfilled any of the following criteria were not eligible for inclusion in this study:

1. Known hypersensitivity to any of the study drugs.

2. Used other investigational drugs at the time of enrolment, or within 30 days or five half-lives of enrolment, whichever was longer; or longer if required by local regulations, and for any other limitation of participation in an investigational trial based on local regulations.

3. Had a history of hypersensitivity to any of the study drugs or to drugs of similar chemical classes.

4. Had a history of clinically significant ECG abnormalities, or any of the following ECG abnormalities at screening or baseline:

- PR >210 msec.
- QRS complex >120 msec.
- QTcF >450 ms or shortened QTcF less than 340 ms or a family history of long QT syndrome or sudden death.
- Second or third degree atrioventricular block.
- Incomplete, full or intermittent bundle branch block.
- Abnormal T wave morphology.

5. Had a history of malignancy of any organ system (other than localised basal cell carcinoma of the skin), treated or untreated, within 5 years previous to enrolling in the study, regardless of whether there was evidence of local recurrence or metastases.

6. Pregnant or nursing (lactating) women.

7. Women of child-bearing potential, defined as all women physiologically capable of becoming pregnant, including women whose career, lifestyle, or sexual orientation precludes intercourse with a male partner and women whose partners have been sterilised by vasectomy or other means.

8. Fertile males, defined as all males physiologically capable of conceiving offspring UNLESS the participant agreed to comply with two highly effective contraceptive methods comprising a barrier method (condom or occlusive cap) for the entire duration of the study, and 120 days following the last study drug administration. Vasectomised males were to use a condom for the entire study and 120 days following drug administration.
9. Smokers (use of tobacco products in the previous 3 months). Urine cotinine levels were to be measured during screening and at each baseline for all participants. Smokers were defined as any participant who reported tobacco use and/or who had a urine cotinine ≥500 ng/mL. For light smokers (defined as less than five cigarettes per day) to pass the cotinine test, smoking should have stopped at least 24 h prior to reporting to the centre (i.e., day -2, early morning). Smoking was not allowed during the study.
10. Use of any prescription drugs, herbal supplements, within 4 weeks prior to initial dosing, and/or over-the-counter (OTC) medication, dietary supplements (vitamins included) within 2 weeks prior to initial dosing (diazepam interferes with the analysis of DSM265 and should not have been used for 8 weeks prior to initial dosing). If needed (i.e., an incidental and limited need), paracetamol was acceptable up 4 g/day, but had to be documented in the concomitant medications/significant non-drug therapies page of the CRF.
11. Intake of grapefruit, grapefruit juice or other products containing grapefruit within 14 days of the first drug administration.
12. Excessive intake of caffeine drinks or energy drinks within 48 h before admission defined as more than three 8 oz. cups of coffee a day, equivalent to roughly 250 mg of caffeine. Defined as 1 (6 oz) cup of coffee: 2 cans of cola: 1 (12 oz) cup of tea: 3 oz milk chocolate.
13. Consumption of broccoli, caviar, sardines, liver, heart and kidneys and yeast supplements one day prior to dosing and throughout the dosing phase. Tomatoes and vegemite were to be avoided 3 h prior to dosing and 3 h post-dose.
14. Donation or loss of 400 mL or more of blood within 8 weeks prior to initial dosing, or longer if required by local regulation.
15. Plasma donation (>100 mL) within 60 days prior to first dosing.
16. Haemoglobin levels below 13·0 g/dL (males) or 11·5 g/dL (females) at screening as determined by full blood cell (FBC) counts.
17. Haptoglobin levels outside reference range for study laboratory (part 1).
18. Positive direct antiglobulin test (direct Coomb's test -DAT) (part 1).
19. ALT and/or AST and/or lactase dehydrogenase (LDH) should be ≤ upper limit of normal (ULN).
20. Liver enzymes other than ALT, AST, LDH elevated ≥1·5 × ULN within 2 weeks prior to initial dosing.
21. Recent (within the last 3 years) and/or recurrent history of autonomic dysfunction (e.g., recurrent episodes of fainting, palpitations, etc.).
22. Recent (within the last 3 years) and/or recurrent history of acute or chronic bronchospastic disease (including asthma and chronic obstructive pulmonary disease, treated or not treated).
23. History of any food allergies.
24. Any surgical or medical condition which might have significantly altered the absorption, distribution, metabolism, or excretion of drugs, or which may have jeopardises the participant in case of participation in the study. The investigator should have made this determination in consideration of the participant's medical history and/or clinical or laboratory evidence of any of the following:

- Inflammatory bowel disease, ulcers, gastrointestinal or rectal bleeding in the last 6 months.
- Major gastrointestinal tract surgery such as gastrectomy, gastroenterostomy, or bowel resection.
- Pancreatic injury or pancreatitis in the last 6 months.
- The investigator should have been guided by the following criteria:
    - *a.* Any single parameter may not have exceeded 1·5 × ULN. A single parameter elevated up to and including 1·5 × ULN should have been re-checked once more as soon as possible, and in all cases, at least prior to enrolment/randomisation, to rule out lab error.
    - *b.* Any elevation of more than one parameter excluded a participant from participation in the study unless otherwise agreed by the medical monitor. Testing may have been repeated once more as soon as possible, but in all cases, at least prior to enrolment/randomisation, to rule out lab error.

Re-check results were required not to be clinically significant in order for participant to qualify and confirmed and agreed by the medical monitor.
25. History or presence of impaired renal function as indicated by clinically significantly abnormal creatinine or urea values, or abnormal urinary constituents (e.g., albuminuria).
26. Evidence of urinary obstruction or difficulty in voiding at screening.
27. History of immunodeficiency diseases, including a positive HIV (ELISA and Western blot) test result.
28. A positive Hepatitis B surface antigen (HBsAg) or Hepatitis C antibody test result.
29. History of drug or alcohol abuse within the 12 months prior to dosing, or evidence of such abuse as indicated by the laboratory assays conducted during screening and/or baseline.

In addition to all of the above exclusion criteria, participants participating in the malaria induced infection phase (part 2) were not to fulfil any of the following exclusion criteria:

1. Had visited a malaria-endemic area for a period greater than 2 weeks in the 12 months previous to study initiation.
2. Lived alone for the duration of the study.
3. Had ever received a blood transfusion.
4. Any clinically significant biochemical or haematologic abnormality (Hb had to be ≥11·5 g/dL females; 13·5 g/dL for males) – including pre-existing red cell antibodies.
5. Had evidence of increased cardiovascular disease risk (defined as >10%, 5 year risk) as determined by the method of Gaziano et al.[1] Risk factors include sex, age, systolic blood pressure (mm Hg), smoking status, body mass index (BMI, kg/m^2), reported diabetes status and blood pressure.
6. Participant was receiving psychiatric drugs or had been hospitalised within the past 5 years prior to enrolment for psychiatric illness, history of suicide attempt or confinement for danger to self or others. Participants who were receiving a single antidepressant drug and were stable for at least 3 months prior to enrolment without decompensating may have been allowed to enrol in the study at the investigator's discretion.
7. History or presence of suicidal behaviour or any suicidal ideation of type 4 or 5 on the Columbia Suicide Severity Rating Scale (C-SSRS) in the past 6 months prior to enrolment or history of suicidality as assessed by the investigator.
8. History of a severe allergic reaction, anaphylaxis or convulsions following any vaccination or infusion.
9. Recent or current therapy with an antibiotic or drug with potential antimalarial activity (tetracyclines, clindamycin, hydroxychloroquine, azithromycin, amodiaquine, sulphonamides, co-trimoxazole, fluoroquinolones etc.).
10. Participants unwilling to defer blood donations to the ARCBS for 6 months after study completion.

Schedule of events

Table S1. Schedule of events part 1: single ascending dose

**Study phase**	**Screening**	**Baseline**	**Treatment period**																	**Study completion[m]**
Visit number[a]	1	2	3													4	5	6	7	8
Study days	Day -28 to -2	Day-1	1											2	3	5	7	10	14	21
Time (h)		-24	Pre-dose	0	0·5	1	2	3	4	6	8	9	12	24	48	96	144	216	312	480
Confinement			x											x	x					
Inclusion/exclusion criteria	x[b]	x[c]																		
Relevant medical history/ current medical conditions	x	x[c]																		
Demography	x																			
Physical examination	x		x[d]												x[d,j]	x[d]	x[d]	x[d]	x[d]	x[d]
Hepatitis & HIV screen	x																			
Alcohol test, drug screen, cotinine	x	x																		
Pregnancy test	x	x																		x
Meal record			x											x	x					
Study completion information																				x
Body height	x																			
Body weight	x	x																		x
Body temperature	x	x	x			x			x		x									
Blood pressure/pulse rate[e]	x[e]	x	x		x[j]	x[j]	x[j]		x[j]	x[j]	x[j]		x[j]	x[j]	x[j]	x	x	x	x	x
ECG evaluation (triplicate)	x	x	x			x[j]			x[j]		x[j]			x[j]						
Cardiac telemetry[f]			x[j]											x[j]						
Haematology, blood chemistry, urinalysis	x	x												x[j]	x[j]	x	x	x	x	x
Microscopy stained blood smears	x	x													x[j]		x		x	
Haemolysis panel	x[g]	x[g]												x[j]	x[j]	x	x[g]	x	x[g]	x[g]
Study drug administration				x																
Pharmacokinetic blood collection[h, i]			x		x[j]	x[j]	x[j]		x[j]	x[j]	x[j]		x[j]	x[j]	x[j]	x	x	x	x	x
Uridine metabolism sample collection[k]			x						x	x			x	x		x				
Urine collection[l]			x											x	x					
Adverse events and serious adverse events	As required																			
Concomitant meds/therapies	As required																			

Table S1. Schedule of events part 1: single ascending dose (continuation)

[a] Visit structure given for internal programming purposes only.
[b] The 250 mg cohort in fed conditions did not have a screening period.
[c] Review of inclusion and exclusion criteria and current conditions was required at baseline evaluation.
[d] Limited physical exam only; focussed on several relevant body systems.
[e] Sitting blood pressure and pulse measurements required.
[f] Cardiac telemetry (25–1200 mg, including fed 250 mg cohort).
[g] Direct antiglobulin test (DAT) were performed at screening, day 1 (before dosing), day 7, day 14, and day 21/end of study.
[h] Pharmacokinetic samples were collected from fasted single dose cohorts (25–1200 mg) and fed single dose cohort (250 mg, same group as 250 mg fasted). The 1200 mg cohort had two extra samples taken at timepoints 648 and 816 h.
[i] Plasma sampling were collected and shipped in parallel for all dry blood spot (DBS) sampling timepoints.
[j] Data from three sentinels (two on DSM265 and one on placebo arm) to be reviewed prior to continuing 25 mg cohort.
[k] Uridine assay samples were collected for the 800 mg and 1200 mg dose-cohorts only.
[l] 150–600 mg cohorts only (including the fed 250 mg cohort).
[m] 1200 mg cohort completed on day 35.

Table S2. Schedule of events part 2: IBSM

Study phase	Screening	Malaria infection and randomisation	Parasite load monitoring		Day 7
Visit number	1	2	3, 4, 5	6, 7, 8, 9	10
**Study day**	**-29 to -1**	**0**	**1 to 3**	**4 to 7**	**7**
Time (h)					
Inclusion/exclusion criteria	x	x[a]			
Demography	x				
Physical examination	x				
Relevant medical history/current medical conditions	x				
Concomitant medication /non-drug therapies	As required				
Hepatitis and HIV screen	x				
Alcohol test, drug screen, urine cotinine	x	x			
Serum storage	x				
Red cell alloantibody	x				
Pregnancy test	x	x			
Concomitant medication /therapies	x				x
**Malaria inoculum application**		x			
Malaria qPCR monitoring and drug resistance[b]		x	x[c]	x[d]	x[d]
Body height	x				
Body weight	x				
Body temperature/blood pressure/pulse rate/respiratory rate	x	x[e]	x[f]	x[f]	x[g]
ECG evaluation (triplicate)	x	x			
Cardiac telemetry					
Haematology, blood chemistry, urinalysis	x	x			
Adverse events and serious adverse events	As required				

Table S2. Schedule of events part 2: IBSM (continuation)

**Study phase**	**Treatment / follow-up period**																**End of study**
	**Study drug treatment and follow-up**												**Artemether-lumefantrine treatment**			**Follow-up visit**	
Visit numbers	11		(12)	14/15	16/17	18	19	20	21	22	23	24	25		26	27	28
**Study day**	**8[h]**	**9**	**10**	**11**	**12**	**13**	**14**	**17**	**19**	**21**	**24**	**25**	**28**	**29**	**30**	**32**	**35**
Time (h)	0	24	48	72	96		144	216		312			480				
Confinement		x															
Inclusion/exclusion criteria	x[a]																
Physical examination	x				x		x				x						x
Hepatitis and HIV screen																	x
Alcohol test, drug screen, urine cotinine	x																
Serum storage																	x
Red cell alloantibody																	x
Pregnancy test	x																x
Concomitant medication/therapies	As required																
Meal record	x		x														
Malaria qPCR monitoring and drug resistance[b]	x[i]	x[d]	x[d]	x[b,d]	x[d]	x[c]	x[c]	x[c]	x[c]	x[b,c]	x[c]	x[c]	x[b,c]		x	x	x[b]
**Drug administration (active/control)**	x																
**Artemether-lumefantrine administration**													x	x	x		
Body temperature	x	x	x	x	x	x	x			x	x						x
Blood pressure/pulse rate/respiratory rate	x				x	x	x			x	x						x
ECG evaluation (triplicate)	x	x											x				x
Cardiac telemetry	x																
Haematology, blood chemistry, urinalysis	x		x							x			x				x
Adverse events and serious adverse events	As required																
Pharmacokinetic blood collection	x[j]				x		x	x		x			x				
Study completion information																	x

Table S2. Schedule of events part 2: IBSM (continuation)

[a] Review of inclusion/exclusion criteria at day 0.
[b] Drug resistance samples.
[c] Sample for qPCR to be collected AM on day 4 only, and nominated days. *pfs25* qRT-PCR collection AM only from day 12 at parasitaemia qPCR timepoints (none day 35)
[d] Samples for qPCR to be collected in 12±4 h time windows, AM and PM.
[e] Body temperature/blood pressure/pulse rate/respiratory rate, to be measured before inoculation and 1 h after inoculation.
[f] Body temperature was to be measured once a day till a participant became qPCR positive and twice a day after participant became qPCR positive.
[g] Body temperature was to be measured twice a day.
[h] Before drug treatment, the level of parasite load was to be confirmed ≥800 parasites/mL. If clinical evidence of malaria occurred or qPCR quantification of ≥2000 parasites/mL was detected before day 0 in the morning, allocated treatment was to begin at that time.
[i] Sample for qPCR was to be collected whilst confined at pre-dose, and at 4, 8, 12, 16, 20, 24 (day 9), 30, 36, and 48 h (day 10) post-dose.
[j] Pharmacokinetic sampling whilst confined was taken pre-dose, and at 0·5, 1, 2, 4, 6, 8, 12, 24, 48, 96 (day 12), 144 (day 14), 216 (day 17), 312 (day 21), 480 (day 28) h post-dose (only for participants dosed with DSM265).

Determination of DSM265 levels in plasma and blood

Blood sampling for pharmacokinetic analysis

Blood samples (4 mL) were collected into sodium heparin tubes. Twenty-µL of blood sample were transferred to a dry blood spot (DBS) card in four replicates. Cards were dried for 2 h at room temperature and individually packed in re-sealable plastic bags. The remainder blood sample was centrifuged at 3–5°C for 10 min at 2000*g*, and the plasma transferred into polypropylene tubes, and stored below -60°C until analysis.

Plasma sample preparation

Plasma samples were centrifuged at 3000 rpm for 5 min and 20 µL of sample was transferred into a 1·5 mL propylene tube or 96 well plate. A volume of 200 µL of methanol was added to each sample tube and vortexed for 1 min. Samples were centrifuged at 2000*g* for 10 min and 25 µL of supernatant transferred into HPLC vials containing 175 µL of Recon solution, vortexed for 1 min, and centrifuged at 2000*g* for 5 min.

Blood sample preparation

DSM265 was extracted from DBS cards by solvent extraction. Samples in DBS cards were cut and transferred to 10 mL polypropylene tubes. A volume of 1 mL of methanol were added to each tube, and gently mixed in a rotatory mixer at 40 rpm for 60 min. Samples were centrifuged at 2000*g* for 5 min and 100 µL of the supernatant transferred to HPLC vials containing 100 µL of Ultra-pure water.

Liquid chromatography/mass spectrometry (HPLC/MS/MS) method

DSM265 blood and plasma concentration was analysed in an HPLC system using a LC-20AD pump (Shimadzu), which was coupled to an API 4000 mass detector (AB Sciex) with a turbo-ion spray source. The mobile phase consisted of 70% of methanol in water for solvent A, and 90% of methanol in water for solvent B, both solvents containing 0·2% of formic acid. A volume of 5 µL was injected at a flow rate of 0·5 mL/min into a C8, 50 × 2 mm, 3 µm column (Phenomenex Luna). Samples were separated with the following gradient: 0 min, 50% solvent B; 2·5 min, 50% solvent B; 2·6 min, 100% solvent B; 3·4 min, 100% solvent B; 3·6 min 0% solvent B; 3·8 min 0% solvent B; 3·9 min 50% solvent B. The total analysis time was 5 min. Protonated ions formed by turbo ion spray in positive mode (MRM) were used to detect DSM265 and the internal standard (diazepam). MS/MS detection was used to monitor the fragmentation of 416·0–396·0 m/z for DSM265, and 284·9–222·2 m/z for the internal standard.

This method was validated by CPR Pharma Services, which is accredited by NATA for compliance with ISO/IEC 17025 (accreditation number: 15130).

Determination of DSM450 levels in plasma

Plasma sample preparation

Blood samples were collected as described for determination of DSM265 levels. DSM450 and the internal standard (carbutamide) were separated from plasma by acetonitrile precipitation. An aliquot of plasma (25–50 µL, sample or spiked standard) was added to 250–275 µL of acetonitrile (containing the internal standard) in a 96-well polypropylene plate. Following vortexing, the plate was centrifuged at 2000*g* for 10 min (4°C). In an automated manner, 100 µL of the supernatant were transferred to a clean 96-well plate containing 100–200 µL of an aqueous solution of 10 mM ammonium formate. Samples were analysed simultaneously with spiked plasma standards.

Liquid chromatography/mass spectrometry (HPLC/MS/MS) method

Several analytical methods were used to detect the DSM450 levels in the different DSM265 dose-cohorts tested. For all dose-cohorts, samples were analysed in an HPLC system using a 1290 Infinity Binary pump (Agilent), which was coupled to a mass detector with a turbo ion-spray interface. The mobile phase consisted of water for solvent A, and acetonitrile for solvent B, both solvents containing 0·1% formic acid. A sample volume of 10 µL was injected into an HPLC column at a flow rate of 0·8 mL/min. After separation, analytes were ionised in the positive ion mode with a source temperature of approximately 550°C. Detection was in the multiple reaction monitoring (MRM) mode at m/z 432·0 to 256·0 for DSM450, and 271·2 to 91·0 for carbutamide.

Dose-cohorts 150 and 250 mg (fasted conditions) were analysed in a Sciex API 5500™ mass detector. Samples were injected into a C18, 30 × 3 mm, 2·7 µm column (Ascentis Express). Samples were separated with the following gradient: 0 min, 30% solvent B; 0·25 min, 65% solvent B; 0·35 min, 70% solvent B; 1 min, 30% solvent B; 2·2 min, 30% solvent B.

Dose-cohorts 400–800 mg, and the 250 mg dose-cohort in fed conditions, were analysed in a Sciex API 6500™ mass detector. Samples were injected into a C18, 30 × 2·1 mm, 5 µm column (Fortis). Samples were separated with the following gradient: 0 min, 5% solvent B; 0·6 min, 98% solvent B; 1·1 min, 5% solvent B; 2·6, 5% solvent B.

Dose-cohort 1200 mg was analysed in a Sciex API 5500™ mass detector. Samples were injected into a C18, 30 × 2·1 mm, 5 µm column (Fortis). Samples were separated with the following gradient: 0 min, 20% solvent B; 0·35 min, 95% solvent B; 0·9 min, 20% solvent B; 1·85 min, 20% solvent B.

Data analysis. DSM450 and internal standard peak areas were determined using Sciex Analyst™ software. The concentration of DSM450 in each sample was calculated by least squares regression analysis of the peak area ratio (DSM450/internal standard) of the spiked human plasma standards versus concentration.

Determination of uridine and uridine nucleotides

Uridine and uridine nucleotides extraction

Blood was collected into 2 mL BD Vacutainer tubes (Becton Dickinson and Company, Franklin Lake, NJ) containing sodium fluoride/Na_2 EDTA (3 mg/6 mg) and stored at -70°C until analysis. Perchloric acid at 2·085% (75 µL, Fisher) was added to blood samples (50 µL), mixed by vortex, and incubated for 10 min. The mix was centrifuged at 16100*g* for 5 min at 4°C. The supernatant (65 µL) was transferred to a fresh tube and centrifuged again under the same conditions. The supernatant (55 µL) was transferred to a new tube and neutralised with base mix (82·5 µL). The base mix consisted of 1·5 mL 350 mM KOH, 1·5 mL 175 nM $KHCO_3$, UMP-internal standard (60 ng/mL), and tolbutamide internal standard (30 ng/mL). Supernatant and base mix were mixed by vortex and centrifuged again as above. The supernatant (100 µL) was collected and mixed with 200 µL of HPLC solvent A (see details in HPLC section below), mixed by vortex and centrifuged as above. The supernatant was transferred to an HPLC vial for analysis.

Liquid chromatography/mass spectrometry (HPLC-MS/MS) method

Blood levels of uridine and uridine nucleotides were analysed in a Shimadzu Prominence HPLC system, which was coupled to a 4000-Qtrap mass detector (AB Sciex, Framingham, MA) with a turbo-ion spray source.
To analyse uridine, the mobile phase consisted of water for solvent A, and acetonitrile for solvent B, both solvents containing 0·2% acetic acid. A volume of 10 µL was injected at a flow rate of 0·4 mL/min into a Synergi Polar –RP, 2·0 × 150 mm, 4 µm column (Phenomenex) with guard column. Samples were separated with the following gradient: 0–0·5 min, 5% solvent B; 0·5–1·5 min, increase to 95% solvent B; 1·5–3·5 min, 95% solvent B; 3·5–3·6 min, decrease to 5% solvent B; 3·6–5·5 min, 5% solvent B. Ions formed by turbo ion spray in negative mode were used to detect uridine and the internal standard tolbutamide. MS/MS detection was used to monitor the fragmentation of uridine from 243·0 to 109·0, and of tolbutamide from 269·1 to 169·9.
To analyse uridine nucleotides, a chromatographic method for nucleotides developed by Hofmann and colleagues was followed.[2] Briefly, the mobile phase consisted of 30% of acetonitrile and 10 mM NH_4 acetate in water for solvent A (pH 6), and 30% of acetonitrile and 1 mM NH_4 acetate in water for solvent B (pH 10·5). A volume of 40 µL was injected at a flow rate of 0·25 mL/min into a Thermo Scientific BioBasic AX, 2·1 × 50 mm, 5 µm column (Fisher) with guard column. Samples were separated with the following gradient: 0–1 min, 0% solvent B; 1–2·5 min, increase to 35% solvent B; 2·5–5 min, 35% solvent B; 5–7 min, increase to 65% solvent B; 7–10 min, 65% solvent B; 10–10·5 min, increase to 100% solvent B; 10·5–15 min, 100% solvent B; 15–15·5 min, decrease to 0% solvent B; 15·5–20·5 min, 0% solvent B. Ions formed by turbo ion spray in positive mode were used to detect uridine nucleotides and the internal standard (UMP-$^{13}C_9$,15N_2; "UMP-IS"). MS/MS detection was used to monitor the fragmentation of UMP from 325·1 to 97·1, of UDP from 405·1 to 97·0, of UTP from 485·0 to 96·9, and of UMP-IS from 336·1 to 102·0.

Determination of uridine and uridine nucleotides concentrations

Standard curves were generated with uridine and uridine nucleotides (UMP, UDP and UTP) standards (Sigma) diluted in blood. The control blood used to prepare the standards was processed at least in triplicate to establish a baseline of endogenous levels of nucleotides. Baseline values were subtracted from the standard curve to generate a standard curve whose ionisation and extraction efficiencies were identical to processed samples. Uridine and uridine nucleotide concentrations in samples were calculated based on subtracted standard curves. Data were collected in duplicate for each sample and averaged prior to analysis. Statistical analysis used two-sample t-test to assess for differences from 0 to 96 h between the groups treated with DSM265 or placebo. Differences in longitudinal changes over the first 96 h between the treatment groups was assessed using a linear mixed model with main effects for time and treatment group and their interaction. A significant interaction term in the linear mixed model would indicate that trends over time differ for the two treatment groups. All analyses were stratified by DSM265 dose-cohorts of 800 mg and 1200 mg (STATA version 13).

Assessment of drug resistance

The presence of *P. falciparum dhodh* (*pfdhodh*) genetic modifications which could confer DSM265 drug resistance was investigated in a recrudescent parasite population (n=5). Blood was taken from participants in part 2 at the time of recrudescence. Parasite DNA was extracted from blood samples, *pfdhodh* gene was amplified by PCR, and its DNA sequence determined.

One of the samples was amplified by PCR using primers 1 and 2 (table S3). The PCR product was directly sequenced with primers 1–8 (table S3).

A lower concentration of parasites in the remainder four samples precluded direct sequencing. For these samples, *pfdhodh* was amplified by PCR using primers 9 and 10 (table S3), subcloned into the cloning vector pGEMT Easy (Invitrogen) and transformed into *E.coli* XL-10 cells. Three clones were sequenced for each construct using primers 1–8 (table S3).

Table S3. *pfdhodh* amplification and sequencing primers

Primer	Sequence
1	ATGATCTCTAAATTGAAACCTC
2	TTAACTTTTGCTATGCTTTCGGCC
3	GTAAATAATAAGAAGGATGTACTTG
4	CCATTCGGTGTTGCTGC
5	GCATTACCCGTTTGGCCCCTTGGGG
6	GCTATTAATGTAAGCTCCCC
7	GGAGGTGTTAGCGGAGC
8	ATCCCTCCTGATGCAATAATGGG
9	GCTCCAGTCGATTTCTTGTTACG
10	TTTGCGCACTTATGTGTCGCCC

DNAStar Seqman was used to compare sample sequences to the canonical 3D7 sequence obtained from PlasmoDB.

Population pharmacokinetic model

The population pharmacokinetic methods and statistical methods were based on the FDA Guidance for Industry Population Pharmacokinetics.[3] The software package NONMEM (ICON Development Solutions, Elicott City, MD, US) was used for the population pharmacokinetic analysis. Model development was carried out using first-order, conditional estimation with interaction (FOCEI). Prior to the population pharmacokinetic analysis, an exploratory analysis of the data was performed using R.

The general model structure characterising the time course of the measured plasma concentrations by dosing group was determined first. Model selection was done on the basis of a Log Likelihood ratio test at an acceptance p-value of 0·01. The difference in two times the Log of the Likelihood (-2LL) between a full and reduced model is approximately asymptotically $\chi 2$ distributed with degrees of freedom equal to the difference in number of parameters between the two models. For example, a decrease of more than 6·63 in -2LL was considered significant at the p<0·01 level for one additional parameter. At each stage of the analysis, the model was evaluated graphically and refined as necessary. Once this process was complete, the resulting model was considered the final population pharmacokinetic model for DSM265.

A one-compartment structural model with first-order absorption was used as the base model. The model fit to the base model was evaluated and appropriate additional models were tested.

Display of adverse events

Table S4. Summary of adverse events following administration of single-doses of DSM265 by system organ class, MedDRA preferred term and dose cohort

	Number (%) of participants with at least one adverse event following treatment with DSM265 or placebo [Number of adverse events]										
**SYSTEM ORGAN CLASS** **Preferred Term**	**DSM265 dose (mg)**										
	**25 mg fasted (n=6)**	**75 mg fasted (n=6)**	**150 mg fasted (n=6)**	**250 mg fasted (n=8)**	**250 mg fed (n=8)**	**400 mg fasted (n=11)**	**600 mg fasted (n=6)**	**800 mg fasted (n=6)**	**1200 mg fasted (n=6)**	**All DSM265 (n=55)**	**Placebo (n=18)**
**INFECTIONS AND INFESTATIONS**	0	1(17%) [1]	0	0	0	2(18%) [2]	1(17%) [1]	1(17%) [1]	0	5(9%) [5]	2(11%) [2]
**Folliculitis**	0	0	0	0	0	1(9%) [1]	0	0	0	1(2%) [1]	0
**Gastroenteritis**	0	0	0	0	0	0	1(17%) [1]	0	0	1(2%) [1]	1(6%) [1]
**Rhinitis**	0	0	0	0	0	1(9%) [1]	0	0	0	1(2%) [1]	0
**Upper respiratory tract infection**	0	1(17%) [1]	0	0	0	0	0	1(17%) [1]	0	2(4%) [2]	1(6%) [1]
**BLOOD AND LYMPHATIC SYSTEM DISORDERS**	0	0	0	0	0	1(9%) [1]	0	0	1(17%) [1]	2(4%) [2]	0
**Thrombocytopenia**	0	0	0	0	0	1(9%) [1]	0	0	1(17%) [1]	2(4%) [2]	0
**METABOLISM AND NUTRITION DISORDERS**	1(17%) [1]	0	0	0	0	0	0	0	0	1(2%) [1]	1(6%) [2]
**Decreased appetite**	1(17%) [1]	0	0	0	0	0	0	0	0	1(2%) [1]	1(6%) [2]
**PSYCHIATRIC DISORDERS**	0	1(17%) [1]	1(17%) [1]	0	0	0	0	0	0	2(4%) [3]	0
**Depression**	0	0	1(17%) [1]	0	0	0	0	0	0	1(2%) [1]	0
**Hypomania**	0	1(17%) [1]	0	0	0	0	0	0	0	1(2%) [1]	0

	Number (%) of participants with at least one adverse event following treatment with DSM265 or placebo [Number of adverse events]										
**SYSTEM ORGAN CLASS**	**DSM265 dose (mg)**										
**Preferred Term**	**25 mg fasted (n=6)**	**75 mg fasted (n=6)**	**150 mg fasted (n=6)**	**250 mg fasted (n=8)**	**250 mg fed (n=8)**	**400 mg fasted (n=11)**	**600 mg fasted (n=6)**	**800 mg fasted (n=6)**	**1200 mg fasted (n=6)**	**All DSM265 (n=55)**	**Placebo (n=18)**
**Insomnia**	0	1(17%) [1]	0	0	0	0	0	0	0	1(2%) [1]	0
**NERVOUS SYSTEM DISORDERS**	3(50%) [4]	2(33%) [3]	3(50%) [5]	5(63%) [7]	4(50%) [5]	3(27%) [5]	1(17%) [3]	0	2(33%) [2]	21(38%) [34]	3(17%) [4]
**Dizziness**	0	0	1(17%) [1]		0	0	0	0	0	1(2%) [1]	1(6%) [1]
**Headache**	2(33%) [2]	2(33%) [2]	3(50%) [4]	5(63%) [7]	4(50%) [5]	3(27%) [5]	1(17%) [3]	0	2(33%) [2]	20(36%) [30]	2(11%) [2]
**Lethargy**	0	0	0	0		0	0	0	0	0	1(6%) [1]
**Paraesthesia**	1(17%) [1]	0	0	0	0	0	0	0	0	1(2%) [1]	0
**Presyncope**	1(17%) [1]	0	0	0	0	0	0	0	0	1(2%) [1]	0
**VIIth nerve paralysis**	0	1(17%) [1]	0	0	0	0	0	0	0	1(2%) [1]	0
**EYE DISORDERS**	0	0	0	1(13%) [1]	1(13%) [1]	0	0	0	0	2(4%) [2]	0
**Blepharospasm**	0	0	0	1(13%) [1]	0	0	0	0	0	1(2%) [1]	0
**Eye pain**	0	0	0	0	1(13%) [1]	0	0	0	0	1(2%) [1]	0
**VASCULAR DISORDERS**	0	1(17%) [1]	0	0	0	0	0	0	0	1(2%) [1]	0
**Flushing**	0	1(17%) [1]	0	0	0	0	0	0	0	1(2%) [1]	0
**RESPIRATORY, THORACIC AND MEDIASTINAL DISORDERS**	0	0	0	0	1(13%) [2]	1(9%) [2]	0	0	1(17%) [2]	3(5%) [6]	2(11%) [2]

	Number (%) of participants with at least one adverse event following treatment with DSM265 or placebo<br>[Number of adverse events]										
**SYSTEM ORGAN CLASS**	**DSM265 dose (mg)**										
**Preferred Term**	**25 mg fasted (n=6)**	**75 mg fasted (n=6)**	**150 mg fasted (n=6)**	**250 mg fasted (n=8)**	**250 mg fed (n=8)**	**400 mg fasted (n=11)**	**600 mg fasted (n=6)**	**800 mg fasted (n=6)**	**1200 mg fasted (n=6)**	**All DSM265 (n=55)**	**Placebo (n=18)**
**Dry throat**	0	0	0	0	0	0	0	0	0	0	1(6%)<br>[1]
**Dyspnoea**	0	0	0	0	0	0	0	0	0	0	1(6%)<br>[1]
**Oropharyngeal pain**	0	0	0	0	1(13%)<br>[1]	1(9%)<br>[1]	0	0	1(17%)<br>[1]	3(5%)<br>[3]	0
**Rhinorrhoea**	0	0	0	0	1(13%)<br>[1]	1(9%)<br>[1]	0	0	0	2(4%)<br>[2]	0
**Sinus congestion**	0	0	0	0	0	0	0	0	1(17%)<br>[1]	1(2%)<br>[1]	0
**GASTROINTESTINAL DISORDERS**	1(17%)<br>[1]	2(33%)<br>[3]	0	2(25%)<br>[2]	0	2(18%)<br>[3]	0	0	2(33%)<br>[3]	9(16%)<br>[12]	0
**Abdominal pain**	0	0	0	0	0	1(9%)<br>[1]	0	0	0	1(2%)<br>[1]	0
**Diarrhoea**	0	1(17%)<br>[1]	0	1(13%)<br>[1]	0	0	0	0	1(17%)<br>[1]	3(5%)<br>[3]	0
**Dry mouth**	0	0	0	0	0	0	0	0	1(17%)<br>[1]	1(2%)<br>[1]	0
**Epigastric discomfort**	0	1(17%)<br>[1]	0	0	0	0	0	0	0	1(2%)<br>[1]	0
**Mouth ulceration**	0	1(17%)<br>[1]	0	0	0	0	0	0	0	1(2%)<br>[1]	0
**Nausea**	0	0	0	1(13%)<br>[1]	0	1(9%)<br>[1]	0	0	0	2(4%)<br>[2]	0
**Vomiting**	1(17%)<br>[1]	0	0	0	0	1(9%)<br>[1]	0	0	1(17%)<br>[1]	3(5%)<br>[3]	0
**SKIN AND SUBCUTANEOUS TISSUE DISORDERS**	2(33%)<br>[2]	0	0	1(13%)<br>[1]	0	0	0	2(33%)<br>[2]	0	5(9%)<br>[5]	1(6%)<br>[1]
**Cold sweat**	0	0	0		0	0	0	0	0	0	1(6%)<br>[1]

	Number (%) of participants with at least one adverse event following treatment with DSM265 or placebo [Number of adverse events]										
**SYSTEM ORGAN CLASS**	**DSM265 dose (mg)**										
**Preferred Term**	**25 mg fasted (n=6)**	**75 mg fasted (n=6)**	**150 mg fasted (n=6)**	**250 mg fasted (n=8)**	**250 mg fed (n=8)**	**400 mg fasted (n=11)**	**600 mg fasted (n=6)**	**800 mg fasted (n=6)**	**1200 mg fasted (n=6)**	**All DSM265 (n=55)**	**Placebo (n=18)**
**Dermatitis contact**	1(17%) [1]	0	0	0	0	0	0	1(17%) [1]	0	2(4%) [2]	0
**Papule**	0	0	0	1(13%) [1]	0	0	0	0	0	1(2%) [1]	0
**Rash**	1(17%) [1]	0	0	0	0	0	0	1(17%) [1]	0	2(4%) [2]	0
**MUSCULOSKELETAL AND CONNECTIVE TISSUE DISORDERS**	2(33%) [3]	1(17%) [1]	1(17%) [1]	1(13%) [1]	1(13%) [1]	0	0	1(17%) [1]	1(17%) [2]	8(15%) [10]	0
**Back pain**	1(17%) [1]	0	0	1(13%) [1]	0	0	0	1(17%) [1]	0	3(5%) [3]	0
**Musculoskeletal chest pain**	0	0	0	0	0	0	0	0	1(17%) [2]	1(2%) [2]	0
**Musculoskeletal stiffness**	1(17%) [1]	0	0	0	0	0	0	0	0	1(2%) [1]	0
**Myalgia**	0	1(17%) [1]	1(17%) [1]	0	1(13%) [1]	0	0	0	0	3(5%) [3]	0
**Pain in extremity**	1(17%) [1]	0	0	0	0	0	0	0	0	1(2%) [1]	0
**GENERAL DISORDERS AND ADMINISTRATION SITE CONDITIONS**	1(17%) [2]	0	0	2(25%) [2]	3(38%) [5]	0	1(17%) [1]	0	2(33%) [2]	8(15%) [12]	2(11%) [3]
**Catheter site bruise**	0	0	0	0	0	0	0	0	1(17%) [1]	1(2%) [1]	0
**Catheter site pain**	0	0	0	1(13%) [1]	0	0	0	0	0	1(2%) [1]	0
**Chest pain**	0	0	0	0	3(38%) [4]	0	0	0	1(17%) [1]	4(7%) [5]	0
**Fatigue**	0	0	0	0	1(13%) [1]	0	0	0	0	1(2%) [1]	2(11%) [3]

	Number (%) of participants with at least one adverse event following treatment with DSM265 or placebo [Number of adverse events]										
**SYSTEM ORGAN CLASS**	**DSM265 dose (mg)**										
**Preferred Term**	**25 mg fasted (n=6)**	**75 mg fasted (n=6)**	**150 mg fasted (n=6)**	**250 mg fasted (n=8)**	**250 mg fed (n=8)**	**400 mg fasted (n=11)**	**600 mg fasted (n=6)**	**800 mg fasted (n=6)**	**1200 mg fasted (n=6)**	**All DSM265 (n=55)**	**Placebo (n=18)**
**Feeling abnormal**	0	0	0	1(13%) [1]	0	0	0	0	0	1(2%) [1]	
**Malaise**	0	0	0	0	0	0	1(17%) [1]	0	0	1(2%) [1]	0
**Vessel puncture site bruise**	1(17%) [2]	0	0	0	0	0	0	0	0	1(2%) [2]	0
**INVESTIGATIONS**	0	0	0	0	1(13%) [1]	1(9%) [1]	0	0	1(17%) [1]	3(5%) [3]	0
**Alanine aminotransferase increased**	0	0	0	0	0	1(9%) [1]	0	0	0	1(2%) [1]	0
**Aspartate aminotransferase increased**	0	0	0	0	1(13%) [1]	0	0	0	0	1(2%) [1]	0
**Reticulocyte count increased**	0	0	0	0	0	0	0	0	1(17%) [1]	1(2%) [1]	0
**INJURY, POISONING AND PROCEDURAL COMPLICATIONS**	0	0	2(33%) [2]	0	0	1(9%) [1]	1(17%) [1]	1(17%) [1]	1(17%) [1]	6(11%) [6]	1(6%) [1]
**Contusion**	0	0	1(17%) [1]	0	0	1(9%) [1]	0	1(17%) [1]	0	3(5%) [3]	0
**Laceration**	0	0	0	0	0	0	0	0	0	0	1(6%) [1]
**Multiple injuries**			1(17%) [1]							1(2%) [1]	
**Muscle strain**	0	0	0	0	0	0	1(17%) [1]	0	0	1(2%) [1]	0
**Thermal burn**	0	0	0	0	0	0	0	0	1(17%) [1]	1(2%) [1]	0

Table S5. Summary of adverse events related to DSM265 treatment by dose cohort

	**Number (%) of participants with at least one suspected drug-related adverse event [Number of adverse events]**										
**Preferred Term**	**DSM265 dose (mg)**										
	**25 fasted (n=6)**	**75 fasted (n=6)**	**150 fasted (n=6)**	**250 fasted (n=8)**	**250 fed (n=8)**	**400 fasted (n=11)**	**600 fasted (n=6)**	**800 fasted (n=6)**	**1200 fasted (n=6)**	**All DSM265 (n=55)**	**Placebo (n=18)**
**Thrombocytopenia**	0	0	0	0	0	0	0	0	1(17%)* [1]	1(2%) [1]	0
**Decreased appetite**	0	1(17%)* [1]	0	0	0	0	0	0	0	1(2%) [1]	
**Headache**		0	1(17%) [1]	3(38%) [3]	1(13%) [1]	2(18%)* [2] (Note: 1 AE grade 1, 1 AE grade 2)	0	0	1(17%) [1]	7(13%) [8]	1(6%) [1]
**Dry mouth**	0	0	0	0	0	0	0	0	1(17%) [1]	1(2%) [1]	0
**Nausea**	0	0	0	0	0	1(9%)* [1]	0	0	0	1(2%) [1]	0
**Vomiting**	0	0	0	0	0	1(9%)* [1]	0	0	0	1(2%) [1]	0
**Malaise**	0	0	0	0	0	0	1(17%)* [1]	0	0	1(2%) [1]	0
**Reticulocyte count increased**	0	0	0	0	0	0	0	0	1(17%)* [1]	1(2%) [1]	0

***** Grade 2 in severity, all other treatment related adverse events were grade 1 in severity. The Common Terminology Criteria for Adverse Events (CTCAE) was used to grade adverse events (grade 1–5). AE: adverse event

Table S6. Summary of adverse events in the IBSM cohort

Adverse events	DSM265 (150 mg)	Mefloquine	Total
Total adverse events	24	6	30
Pre-inoculum	0	0	0
Post-inoculum/pre-DSM265 or mefloquine	5	0	5
Post-DSM265/pre-artemether-lumefantrine	16	0	16
Post-mefloquine	NA	6	6
Post-artemether-lumefantrine	3	NA	3
Probably related to inoculum	16	2	18
Probably related to DSM265	0	NA	0
Probably related to mefloquine	NA	0	0
Probably related to artemether-lumefantrine	0	NA	0

Values indicate the number of adverse events reported. NA: not applicable

Table S7. Probably related adverse events in the IBSM cohort

Category	Event	DSM265 (150 mg)	Mefloquine	Total
**Symptoms**	Fatigue	1	1	2
	Headache	8	0	8
	Myalgia	2	0	2
**Signs**	Chills	1	0	1
	Fever	4	1	5
**Total number of adverse events**		16	2	18

All probably related adverse events reported in the IBSM cohort were related to the inoculum. No adverse events related to DSM265 (150 mg) were reported. Values indicate the number of adverse events reported.

Parmacokinetic analysis

Table S8. Blood pharmacokinetic variables of DSM265 by dose cohort (fasted conditions)

**Dose (mg)**	**C_{max} (ng/mL)**	**t_{max} (h)**	**C_{168} (ng/mL)**	**$AUC_{0-\infty}$ (ng.h/mL)**	**$t_{1/2}$ (h)**
25 (n=6)	683 (33)	2 (1–4)	110 (30)	54200 (23)	87 (27)
75 (n=6)	2020 (35)	1·5 (0·5–4)	291 (40)	156000 (25)	89 (30)
150 (n=6)	3710 (29)	2 (1–2)	669 (37)	341000 (32)	104 (20)
250 (n=8)	5660 (27)	2 (1–2)	1080 (27)	526000 (25)	110 (29)
400 (n=5)	6410 (35)	4 (1·1–12·1)	1540 (37)	712000 (37)	92 (27)
600 (n=6)	8500 (23)	4 (2–4)	2570 (26)	1180000 (25)	114 (15)
800 (n=6)	9280 (25)	3 (2–8)	2670 (46)	1270000 (37)	93 (48)
1200 (n=6)	16700 (37)	2 (0·5–4)	4530 (26)	2130000 (19)	116 (39)

Data are geometric means (coefficient of variation) or for t_{max} median (range). C_{max}: peak blood concentration; t_{max}: timepoint at which C_{max} is reached; C_{168}: DSM265 concentration 168 h post-dose; $AUC_{0-\infty}$: area under the concentration-time curve from 0 h to infinity. t½ : estimated elimination half-life. For the 400 mg dose cohort, only results of the repeated dose cohort are presented.

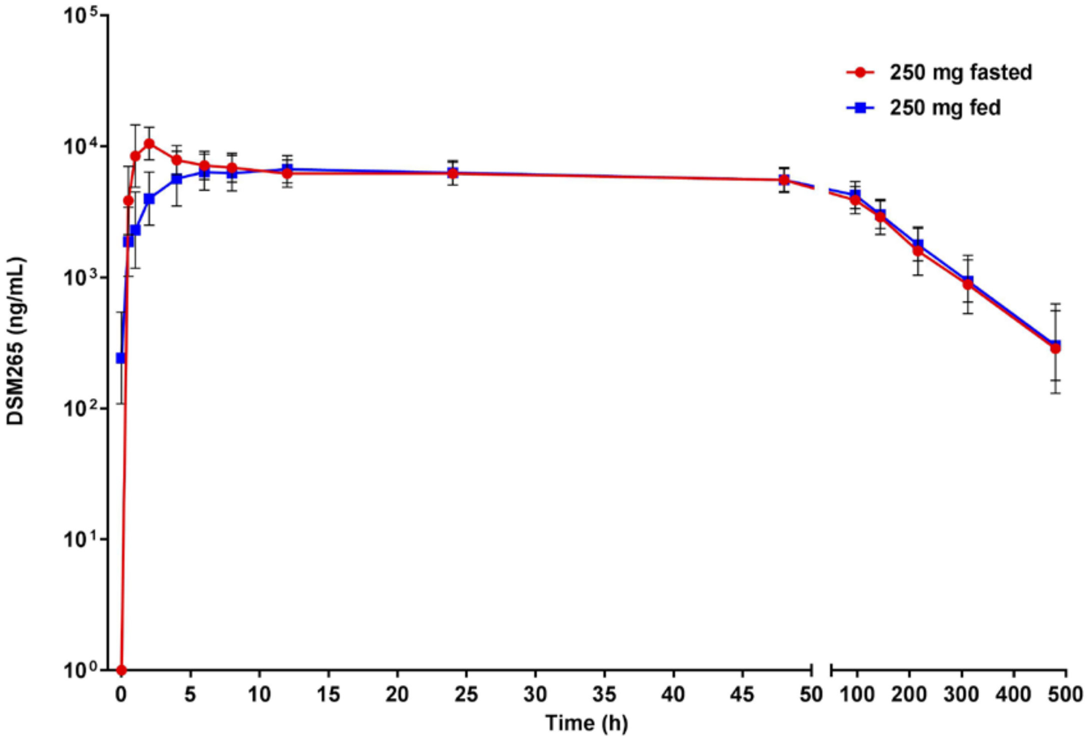

Figure S1. Mean plasma concentration following administration of DSM265 (250 mg) under fasted and fed conditions. Geometric mean plasma concentration of participants in the 250 mg dose cohort in fasted (n=8) and fed conditions (n=8). Time 0 h corresponds to time of DSM265 administration. Error bars represent standard deviations.

Table S9. Plasma pharmacokinetic variables of DSM265 dose cohort 250 mg in fasted and fed conditions (FDA high-fat breakfast)

**Parameter**	**Fasted (n=8)**	**Fed (n=8)**
C_{max} (ng/mL)	11900 (28)	7270 (27)
t_{max} (h)	2 (1– 2)	10 (6–24)
$AUC_{0-\infty}$ (h.ng/mL)	1130000 (25)	1150000 (23)
C_{168h} (ng/mL)	2380 (29)	2540 (25)
$t_{1/2}$ (hr)	104 (27)	103 (22)

Data are geometric means (coefficient of variation) or median (range) for t_{max}.

Table S10. Plasma pharmacokinetic variables of DSM450 by dose cohort

Dose (mg)	C_{max} (ng/mL)	t_{max} (h)	AUC_{480} (h.ng/mL)	$AUC_{0-\infty}$ (h.ng/mL)	$t_{1/2}$ (h)
**Fasted conditions**					
150 (n=6)	471 (45)	95 (95–214)	139000 (48)	167000 (47)	154 (14)
250 (n=8)	687 (39)	95 (48–213)	198000 (43)	217000 (51)	138 (25)
400 (n=6)	1070 (38)	96 (94–311)	292000 (45)	271000 (24)	117 (17)
600 (n=6)	1380 (72)	143 (94–215)	390000 (67)	477000 (75)	164 (31)
800 (n=6)	1920 (35)	96 (48–144)	490000 (38)	454000 (38)	98 (62)
1200 (n=6)	3070 (63)	143 (95–215)	935000 (50)	1170000 (47)	156 (47)
**Fed conditions**					
250 (n=8)	701 (45)	96 (95–144)	187000 (41)	217000 (42)	142 (28)

Data are geometric means (coefficient of variation) or for t_{max} median (range). C_{max}: peak plasma concentration; t_{max}: timepoint at which C_{max} is reached; AUC_{480}: area under the concentration time curve from 0 to 480 h post-dosing; $AUC_{0-\infty}$: area under the concentration-time curve from 0 h to infinity. t½ : estimated elimination half-life. For the 400 mg dose cohort, only results for the repeated dose cohort are presented.

Table S11. Blood pharmacokinetic variables of DSM265 (150 mg) in part 1 (single ascending dose) and part 2 (IBSM)

**Parameter**	**Part 1 (single ascending dose)**	**Part 2 (IBSM)**
C_{max} (ng/mL)	3710 (29)	3885 (26)
t_{max} (h)	2 (1–2)	2 (1–6)
$AUC_{0-\infty}$ (h.ng/mL)	341000 (32)	359644 (9)
$t_{1/2}$ (h)	104 (20)	99 (23)

Data are geometric means (coefficient of variation) or for t_{max} median (range)

Table S12. Population pharmacokinetic parameters of DMS265 in plasma

Parameter	Mean	SE	RSE (%)
**Fixed effects (theta)**			
V (L)	7·68	2·74	35·7
CL (L/h)	0·24	0·04	15·1
D1 (h) (≤250 mg)	1·84	0·17	9·1
D1 (h) (≥400 mg)	3·51	0·46	13·0
V2 (L)	23·4	6·49	27·7
CL2 (L/h)	11·8	6·50	55·1
F1 (400 mg)	0·87	0·98	112·5
F1 (600 mg)	0·75	0·13	16·8
F1 (800 mg)	0·62	0·10	16·7
**Participant random effect (ETA)**			
V (L)	0·4140	0·3510	84·8
CL (L/h)	0·0447	0·1480	331·1
F1	0·0410	0·0434	105·9
V3 (L/h)	0·1440	0·0874	60·7
**Residual variability (sigma)**			
Proportional error (CV%)	0·0296	0·0041	13·7

V: central volume; CL: central clearance ; D1: duration of zero-order absorption; V2: peripheral volume; CL2: peripheral clearance; F1: bioavailability relative to doses; V3: deep tissue volume; SE: standard error; RSE: relative standard error (parameter/SE × 100).

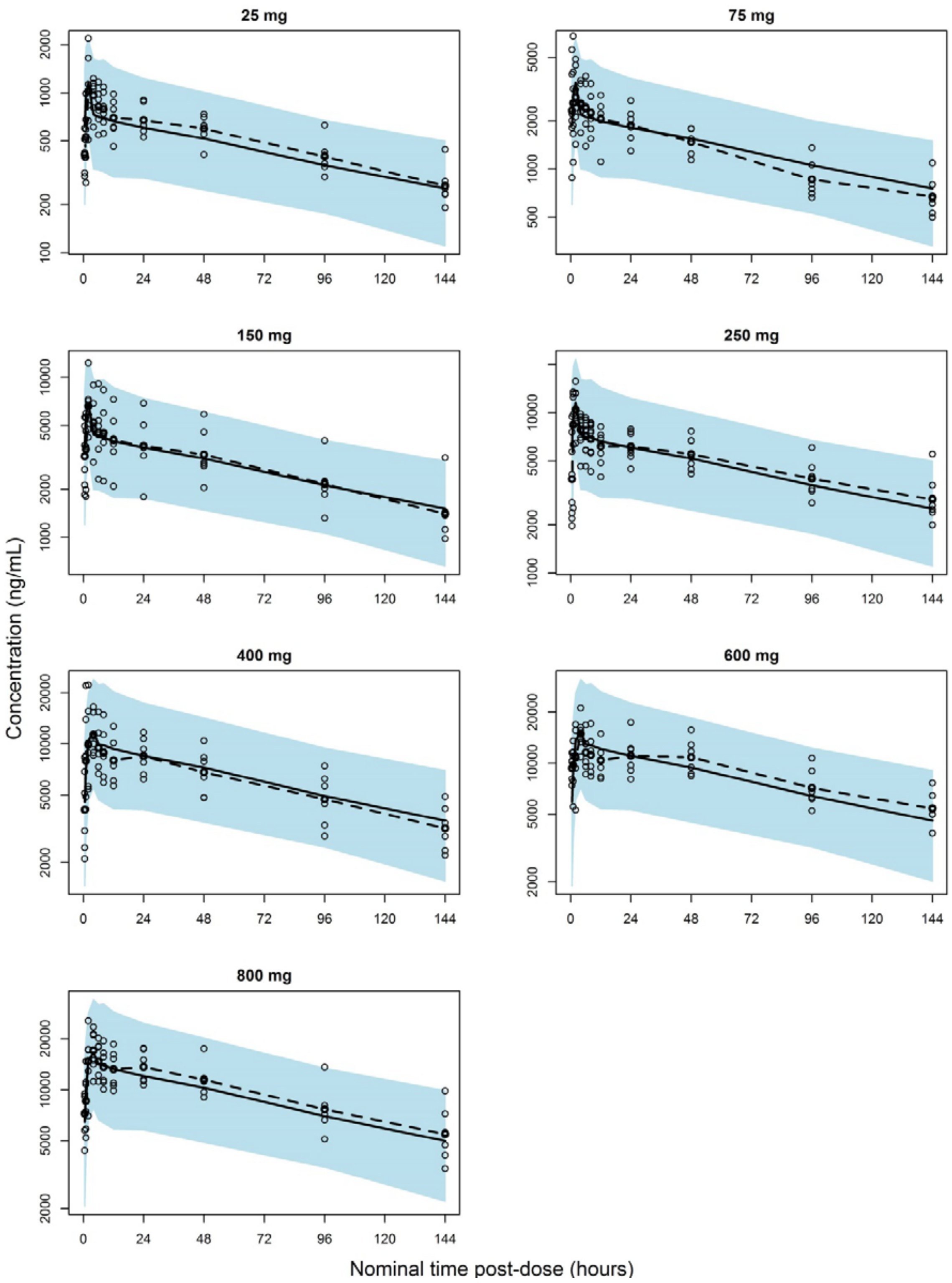

Figure S2. Population pharmacokinetic analysis visual predictive check by DSM265 dose cohort. Observed (open circles) and geometric mean (dashed line) plasma concentrations over nominal time post-dose by dosing cohort overlaid by the median and spread (2·5th to 97·5th percentile) of the predicted concentrations (solid line and blue shading, respectively).

Parasitaemia and gametocytaemia levels in the IBSM cohort

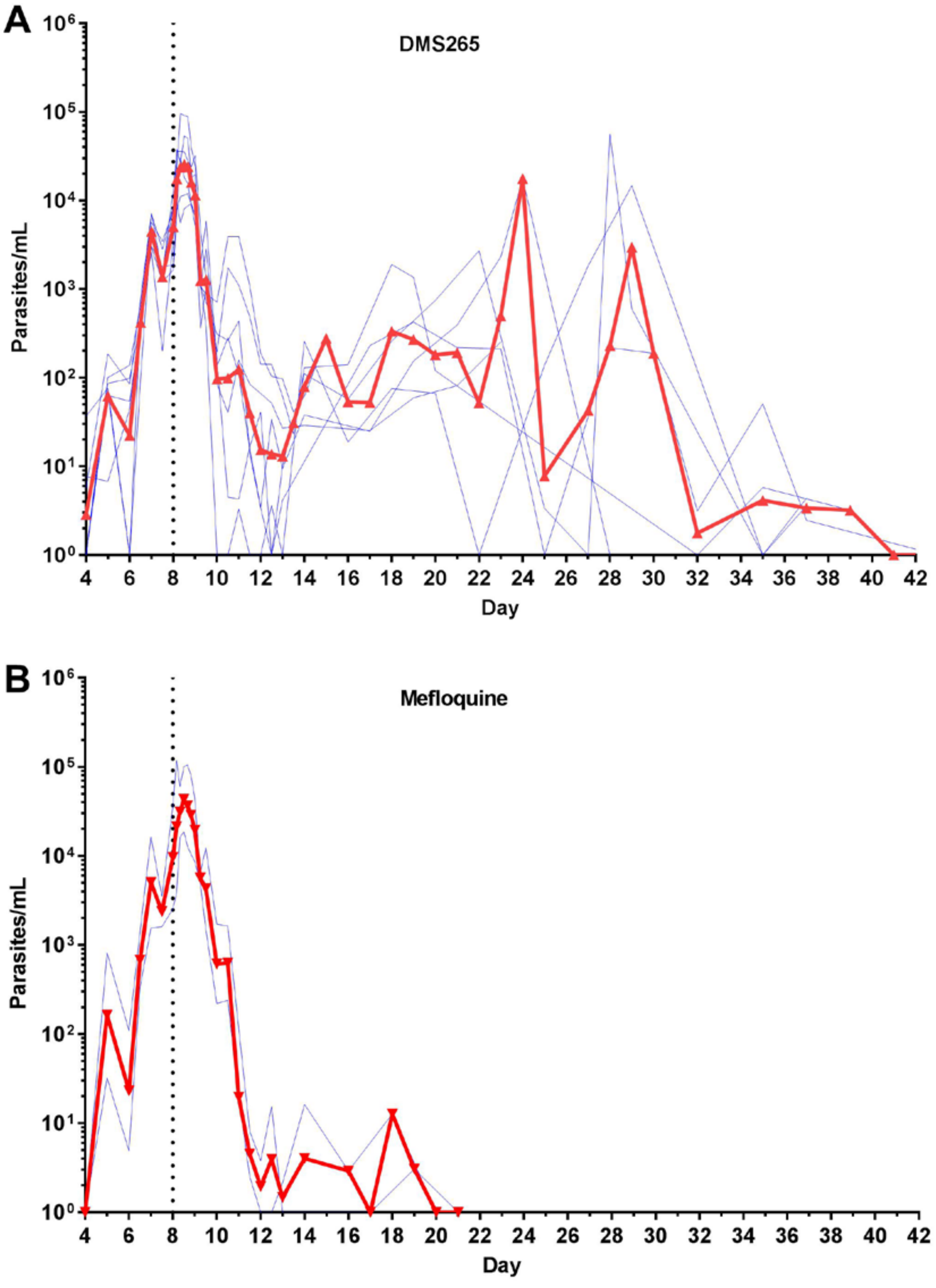

Figure S3. Parasite clearance profiles. Individual parasitaemia level (blue lines) before and after drug treatment with (A) DSM265 (150 mg) or (B) mefloquine (10 mg/kg). Participants were inoculated on day 0 and treated on day 8 (vertical dashed line). Red lines represents mean parasitaemia.

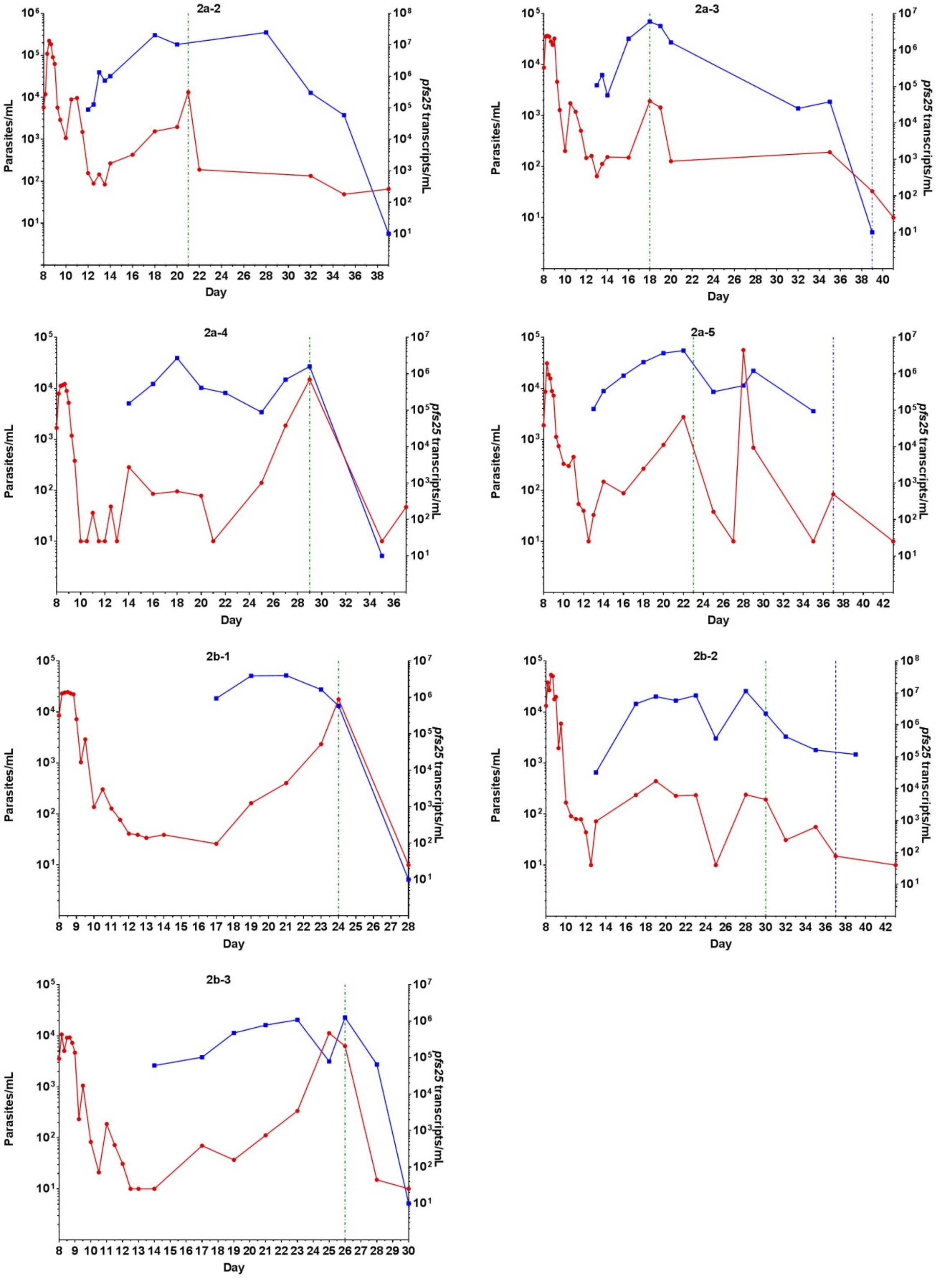

Figure S4. Individual parasitaemia and gametocytaemia levels in the IBSM cohort following administration of DSM265 (150 mg). Parasitaemia (red, left y-axis) and gametocytaemia (blue, right y-axis) levels for each participant in part 2a (2a-1 to 2a5) and part 2b (2b-1 to 2b-3). Participants were inoculated on day 0 and treated with DSM265 (150 mg) on day 8. Time of treatment with artemether-lumefantrine is represented with a green vertical line, and with primaquine with a blue vertical line.

Parasite reduction ratio in the IBSM cohort

Table S13. $Log_{10}PRR_{48}$ and parasite clearance half-lives

Treatment administered	$Log_{10}PRR_{48}$ (95% CI)	Parasite clearance half-life (95% CI) (h)
DSM265 150 mg (n=6)	1·55 (1·42–1·67)	9·4 (8·7–10·2)
Part 2a - DSM265 150 mg (n=3)	1·34 (1·19–1·49)	10·8 (9·7–12·1)
Part 2b - DSM265 150 mg (n=3)	2·04 (1·81–2·28)	7·1 (6·3–8·0)
Mefloquine (n=2)	2·34 (2·17–2·52)	6·2 (5·7–6·7)

$Log_{10}PRR_{48}$ and parasite clearance half-life estimates for the subgroups were derived from the weighted mean of the optimal slope of the log-linear relationship of the parasitaemia decay for participants within each group with significant regression models.

Table S14. Participant-specific $log_{10}PRR_{48}$ estimates

**Participant**	**$Log_{10}PRR_{48}$ (95% CI)**
**DSM265**	
Part 2a	
2a-2	1·10 (0·84–1·37)
2a-3	1·13 (0·90–1·37)
2a-4	*4·62 (2.99–6.26)*
2a-5	1·95 (1·66–2·25)
Part 2b	
2b-1	2·07 (1·78–2·36)
2b-2	2·17 (1.67–2·67)
2b-3	1·76 (1.15–2·37)
**Mefloquine**	
2a-1 (part 2a)	2·43 (2·21–2·66)
2b-4 (part 2b)	2·22 (1·95–2·48)

Participants in italics did not have significant optimal regression fits (p-value >0·001) and were not included in the $log_{10}PRR_{48}$ estimates presented in table 5 of the manuscript.

Human DHODH activity

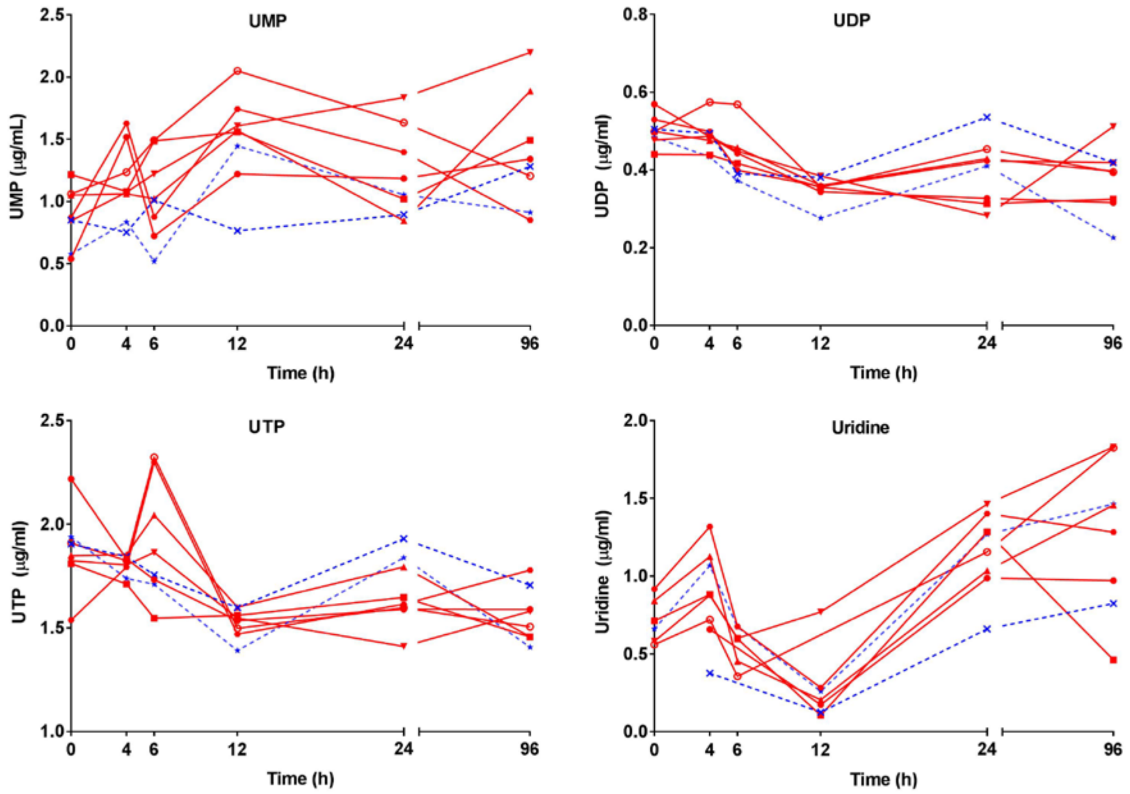

Figure S5. Uridine and uridine nucleotide levels after treatment with DSM265 (800 mg). Individual uridine and uridine nucleotide levels after treatment with 800 mg of DSM265 (red, n=6) or placebo (blue, n=2). Time = 0 correspond with time of treatment with DSM265 or placebo.

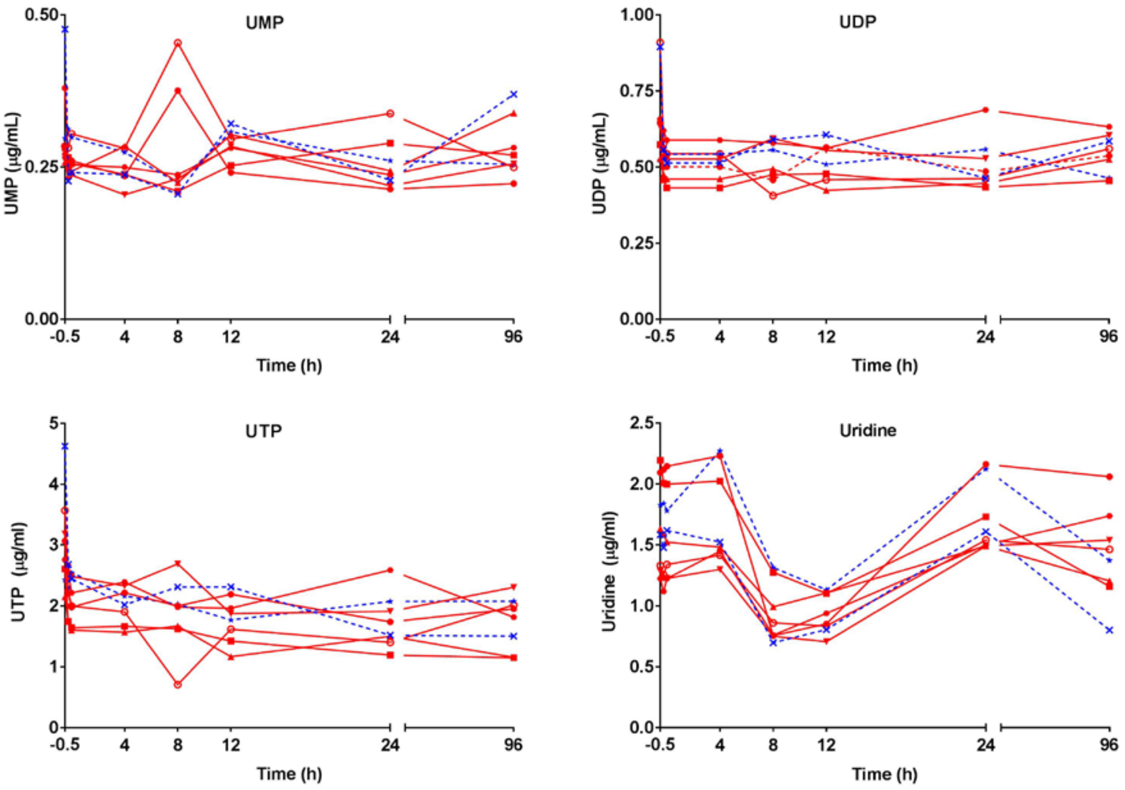

Figure S6. Uridine and uridine nucleotide levels after treatment with DSM265 (1200 mg). Individual uridine and uridine nucleotide levels following treatment with 1200 mg of DSM265 (red, n=6) or placebo (blue, n=2). Time = 0 correspond with time of treatment with DSM265 or placebo.

Table S15. Comparison of changes in uridine and uridine nucleotides levels from 0 to 96 h between DSM265 (800 mg) and placebo groups

Biomarker	Placebo mean changes from 0 to 96 h (95% CI) (μg/mL) (n=2)	DSM265 800 mg mean changes from 0 to 96 h (95% CI) (μg/mL) (n=6)	p-value
UMP	0·382<br>(95% CI -0·225–0·989)	0·565<br>(95%CI 0·020–1·110)	0·44
UDP	-0·172<br>(95% CI -1·271–0·926)	-0·108<br>(95%CI -0·204–-0·013)	0·59
UTP	-0·365<br>(95%CI -2·478–1·748)	-0·297<br>(95%CI -0·603–0·009)	0·77
Uridine	-<br>[n=1]	0·649<br>(95%CI -0·144–1·442) [n=5]	-

No comparison could be made for uridine levels because only data from one placebo participant was available at timepoint 0 h. Please note that for uridine levels the DSM265 sample consisted of five of the six participants. Data represents the mean and 95% CI of the change from 0 to 96 h. Differences between the two treatment groups was assessed by a two-sample t-test at 5% significance level. UMP: uridine monophosphate; UDP: uridine diphosphate; UDP: uridine triphosphate.

Table S16. Comparison of changes in uridine and uridine nucleotides levels from 0 to 96 h between DSM265 (1200 mg) and placebo groups

Biomarker	Placebo mean changes from 0 to 96 h (95% CI) (μg/mL) (n=2)	DSM265 1200 mg mean changes from 0 to 96 h (95% CI) (μg/mL) (n=6)	p-value
UMP	0·042<br>(95% CI -1·057–1·141)	0·011<br>(95% CI -0·046–0·066)	0·78
UDP	-0·003<br>(95% CI -0·956–0·950)	0·044<br>(95% CI 0·019–0·069)	0·64
UTP	-0·706<br>(95% CI -3·725–2·313)	-0·255<br>(95% CI -0·489–0·021)	0·28
Uridine	-0·613<br>(95% CI -3·228–2·002)	-0·048<br>(95% CI -0·558–0·461)	0·14

Data represents the mean and 95% CI of the change from 0 to 96 h. Differences between the two treatment groups was assessed by a two-sample t-test at 5% significance level. UMP: uridine monophosphate; UDP: uridine diphosphate; UDP: uridine triphosphate.

Table S17. Comparison of longitudinal changes in uridine and uridine nucleotides levels over time between DSM265 (800 mg) and placebo groups

	UMP		UDP		UTP		Uridine	
	**Coef**	**p-value**	**Coef**	**p-value**	**Coef**	**p-value**	**Coef**	**p-value**
**Intercept**	0·83	<0·001	0·44	<0·001	1·79	<0·001	0·55	0·001
**Treatment**	0·38	0·008	0·009	0·80	0·004	0·97	0·14	0·46
**Time**	0·003	0·30	-0·001	0·06	-0·002	0·19	0·006	0·06
**Time × treatment**	0·0003	**0·93**	0·0004	**0·58**	<0·0001	**0·82**	<0·0001	**0·91**
**Random effects variance**	<0·001		0·001		<0·001		0·010	
**Residual error**	0·12		0·005		0·04		0·14	

A linear mixed model was used to assess for differences in changes over time between the DSM265(800 mg) and placebo groups, where "coef" is the estimated model coefficient. The interaction effect between the time and treatment group were insignificant (bold p-values, 5% significance level). UMP: uridine monophosphate; UDP: uridine diphosphate; UDP: uridine triphosphate.

Table S18. Comparison of longitudinal changes in uridine and uridin e nucleotides levels over time between DSM265 (1200 m)g and placebo groups

	UMP		UDP		UTP		Uridine	
	**Coef**	**p-value**	**Coef**	**p-value**	**Coef**	**p-value**	**Coef**	**p-value**
**Intercept**	0·28	<0·001	0·57	<0·001	2·45	<0·001	1·58	<0·001
**Treatment**	-0·01	0·55	-0·03	0·49	-0·36	0·20	-0·16	0·47
**Time**	<0·0001	0·65	<0·0001	0·35	-0·01	0·04	-0·005	0·11
**Time × treatment**	<0·0001	**0·71**	<0·0001	**0·43**	0·004	**0·46**	0·006	**0·11**
**Random effects variance**	0·0001		0.002		0·07		0·05	
**Residual error**	0·003		0·007		0·28		0·15	

A linear mixed model was used to assess for differences in changes over time between the DSM265(1200 mg) and placebo groups, where "coef" is the estimated model coefficient. The interaction effect between the time and treatment group were insignificant (bold p-values, 5% significance level). UMP: uridine monophosphate; UDP: uridine diphosphate; UDP: uridine triphosphate.

References

1. Gaziano TA, Young CR, Fitzmaurice G, Atwood S, Gaziano JM. Laboratory-based versus non-laboratory-based method for assessment of cardiovascular disease risk: the NHANES I follow-up study cohort. *Lancet* 2008; **371**(9616): 923–31.
2. Hofmann U, Heinkele G, Angelberger S, et al. Simultaneous quantification of eleven thiopurine nucleotides by liquid chromatography-tandem mass spectrometry. *Anal Chem* 2012; **84**(3): 1294–301.
3. Food and Drug Administration (FDA). Guidance for industry: population pharmacokinetics. Rockville, 1999. http://www.fda.gov/downloads/Drugs/.../Guidances/UCM072137.pdf (accessed Decemer 15, 2016).
